# Supplementary material for: A general assay platform to study protein pharmacology using ligand-dependent structural dynamics
Source: Nat Commun. 2025 May 10;16:4342. doi: 10.1038/s41467-025-59658-6 (PMC12064818; doi:10.1038/s41467-025-59658-6)
Supplement: Supplementary file 1 — Supplementary Information [file 41467_2025_59658_MOESM1_ESM.pdf]

# A general strategy to study protein pharmacology using ligand-dependent structural dynamics

Daniel A. Ciulla[1*], Patricia K. Dranchak[1*], Mahesh Aitha[1], Renier H. P. van Neer[1], Divia Shah[1], Ravi Tharakan[1], Kelli M. Wilson[1], Yuhong Wang[1], John C. Braisted[1] and James Inglese[1,2,§]

[1]Division of Preclinical Innovation, National Center for Advancing Translational Sciences, NIH, Rockville, MD

[2]Metabolic Medicine Branch, National Human Genome Research Institute, NIH, Bethesda, MD

**Supplementary Figure** **Page**

**Supplementary Figure 1.** FLuc and NLuc enzyme mechanisms………. 3

**Supplementary Figure 2.** FLuc inhibitors / analogs library and output summary........................................................................................ 4

**Supplementary Figure 3.** SAR correlation for enzyme and SDR assay activity from FLuc library clade P…………………………………….. 5

**Supplementary Figure 4**. SAR correlation for enzyme and SDR assay activity from FLuc library clade O……………….....……………….. 6

**Supplementary Figure 5**. Expanded clade S evaluated for ATP dependence with SDR and functional enzyme assays……………………….. 7-10

**Supplementary Figure 6**. Comparison of SDR and Thermofluor analysis of select clade S analogs…...………………………………...…….. 11

**Supplementary Figure 7**. Limit of detection of basal and ligand-mediated ABL1-*N*-HiBiT SDR output……………………………………………… 12

**Supplementary Figure 8.** Heatmap displaying ABL1 and PKA pIC50 or $pSDR_{50}$ for 128 kinase inhibitors ………………………………… 13

**Supplementary Figure 9.** ABL1-*N*-HiBiT enzyme inhibition and SDR assay activity…………………………………………………….. 14-18

**Supplementary Figure 10.** Allosteric ligand effect on ABL1-*N*-HiBiT enzyme activity ………………………………………………………… 19

**Supplementary Figure 11.** PKA-*N*-HiBiT enzyme inhibition and SDR assay activity ........................................................................................ 20-24

**Supplementary Figure 12.** Limit of detection of basal and ligand-mediated iPGM-*C*-HiBiT SDR output ……………….................................... 25

**Supplementary Figure 13.** Estimation of DHFR-*C*-HiBiT concentration from cellular lysate………………………………………….................... 26

**Supplementary Figure 14.** Basal RLU range from HiBiT tagged proteins 27

**Supplementary Figure 15**. Correlation between FLuc-*C*-HiBiT SDR % activity (max. response) vs. $pSDR_{50}$ across select compounds sets..... 28

**Supplementary Figure 16**. SDS-PAGE analysis of recombinant *E. coli* expressed and purified proteins. ..........................……………………..... 29

# A general strategy to study protein pharmacology using ligand-dependent structural dynamics

Daniel A. Ciulla[1*], Patricia K. Dranchak[1*], Mahesh Aitha[1], Renier H. P. van Neer[1], Divia Shah[1], Ravi Tharakan[1], Kelli M. Wilson[1], Yuhong Wang[1], John C. Braisted[1] and James Inglese[1,2,§]

[1]Division of Preclinical Innovation, National Center for Advancing Translational Sciences, NIH, Rockville, MD

[2]Metabolic Medicine Branch, National Human Genome Research Institute, NIH, Bethesda, MD

**Supplementary Table** **Page**

**Supplementary Table 1.** DNA ligase ligand binding parameters….............. 30
**Supplementary Table 2.** PubChem Accession AIDs................................. 31
**Supplementary Table 3.** Protocol for Thermofluor assay………………….. 32
**Supplementary Table 4**. Protocol for Firefly luciferase (FLuc) and NanoLuc (NLuc) enzymatic luminescence assay………………....………… 33
**Supplementary Table 5**. Protocol for Abelson kinase (ABL1) and protein kinase A (PKA) peptide phosphorylation assays………………..… 34
**Supplementary Table 6**. Protocol for Co-factor independent phosphoglycerate mutase (iPGM) coupled-enzyme assay…..…………….….…….. 35
**Supplementary Table 7**. Protocol for Co-factor independent phosphoglycerate mutase (iPGM) fluorescent polarization competition-biding assay.… 36
**Supplementary Table 8.** Protocol for DNA ligase activity agarose gel assay… 37
**Supplementary Table 9.** Protocol for Dihydrofolate reductase (DHFR) enzymatic absorbance assay……………………………………… 38
**Supplementary Table 10.** Protocol for SDR assay non-aqueous ligand and aqueous ligand dispense …………………………………………… 39
**Supplementary Table 11.** SDR assay buffer and reader settings ............ 40

## Additional Supplementary Information

**Supplementary Equations**...…………………………………………………….. 41
**Uncropped gels**…………………..…………………………………………….. 42-44
**Supplementary HRMS Data** ..……..……………………………………………. 45-51
**Supplementary** HRMS analysis protocol ….…..………………………………….. 52

**Supplementary Figure 1.** FLuc and NLuc enzyme mechanisms

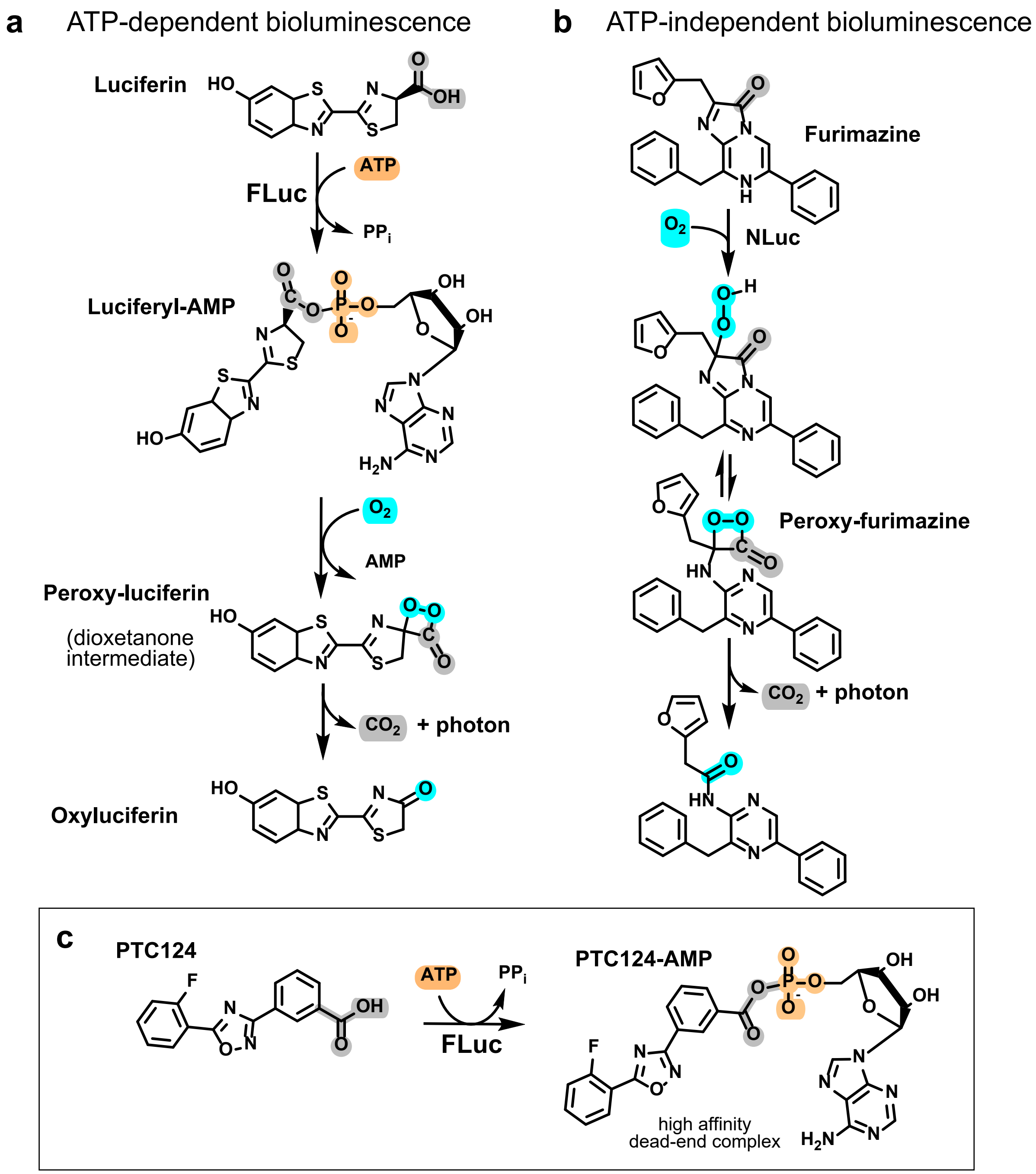


**Supplementary Figure 1. FLuc and NLuc enzyme mechanisms. a**, FLuc, a bioluminescent enzyme, catalyzes the *ATP-dependent* adenylation of the carboxylic acid, luciferin to generate the acyl adenylate, luciferyl-AMP, that in the presence of molecular oxygen undergoes oxidation to a high energy dioxetanone intermediate. Subsequent dioxetanone decomposition generates oxyluciferin, $CO_2$, AMP and photon. **b**, NLuc is *ATP-independent* where following enzyme mediated furimazine oxidation, subsequent decomposition of the cyclic peroxide releases $CO_2$ and generates a photon. **c**, The initial FLuc adenylation reaction can be inhibited by ligands that bind to either the luciferin substrate (*e.g.*, PTC124) or ATP cofactor binding sites or across both (*e.g.*, PTC124-AMP). Supports **Figure 1**.

**Supplementary Figure 2.** FLuc inhibitors / analogs library and output summary

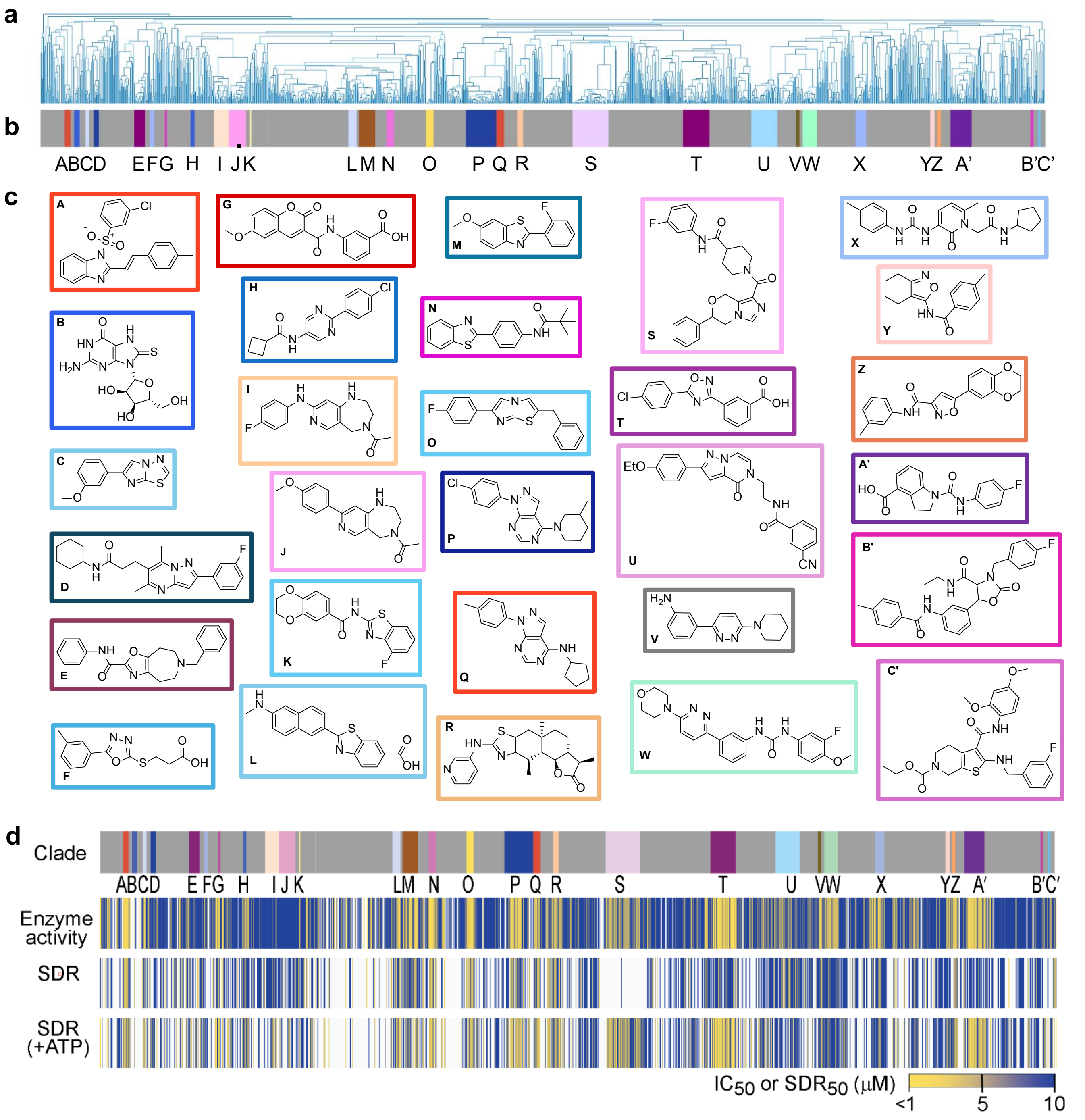


**Supplementary Figure 2. FLuc inhibitors / analogs library and output summary**. **a**, Chemotype hierarchical clustering based on Tanimoto (TT) similarity (top), **b**, clades of interest (color labeled bars) and **c**, representative compound structures. **d**, Activity heatmap for enzyme activity and conditional SDR activity (*e.g.*, ±ATP, luciferin analog). Letters represent clades containing compounds with a TT similarity >0.8. See **Supplementary Data 1** and AIDs 1963320, 1963319 and 1963318 for source data.

**Supplementary Figure 3.** SAR correlation for enzyme and SDR assay activity from FLuc library clade P

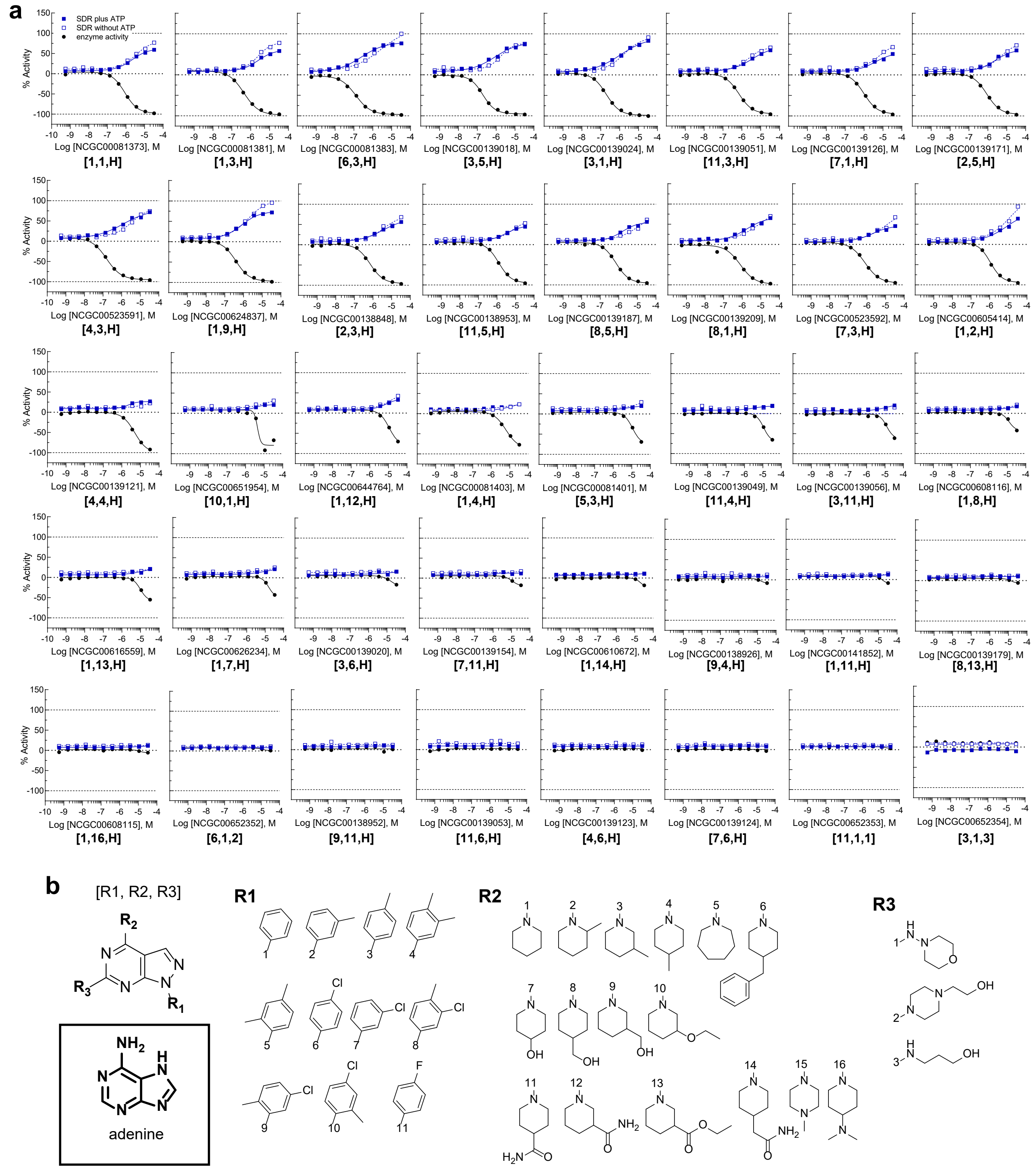


**Supplementary Figure 3. SAR correlation for enzyme and SDR assay activity from FLuc library clade P.** **a**, Concentration response curves for the pyrazolo pyrimidine series (clade P), where [R1, R2, R3] indicate the respective points of attachment of R-groups to core. **b**, Pyrazolo pyrimidine core and R-group members (R1, R2 R3), with similarity to adenine (box). See **Supplementary Data 2** and AIDs 1963320, 1963319 and 1963318 for source data.

**Supplementary Figure 4**. SAR correlation for enzyme and SDR assay activity from FLuc library clade O

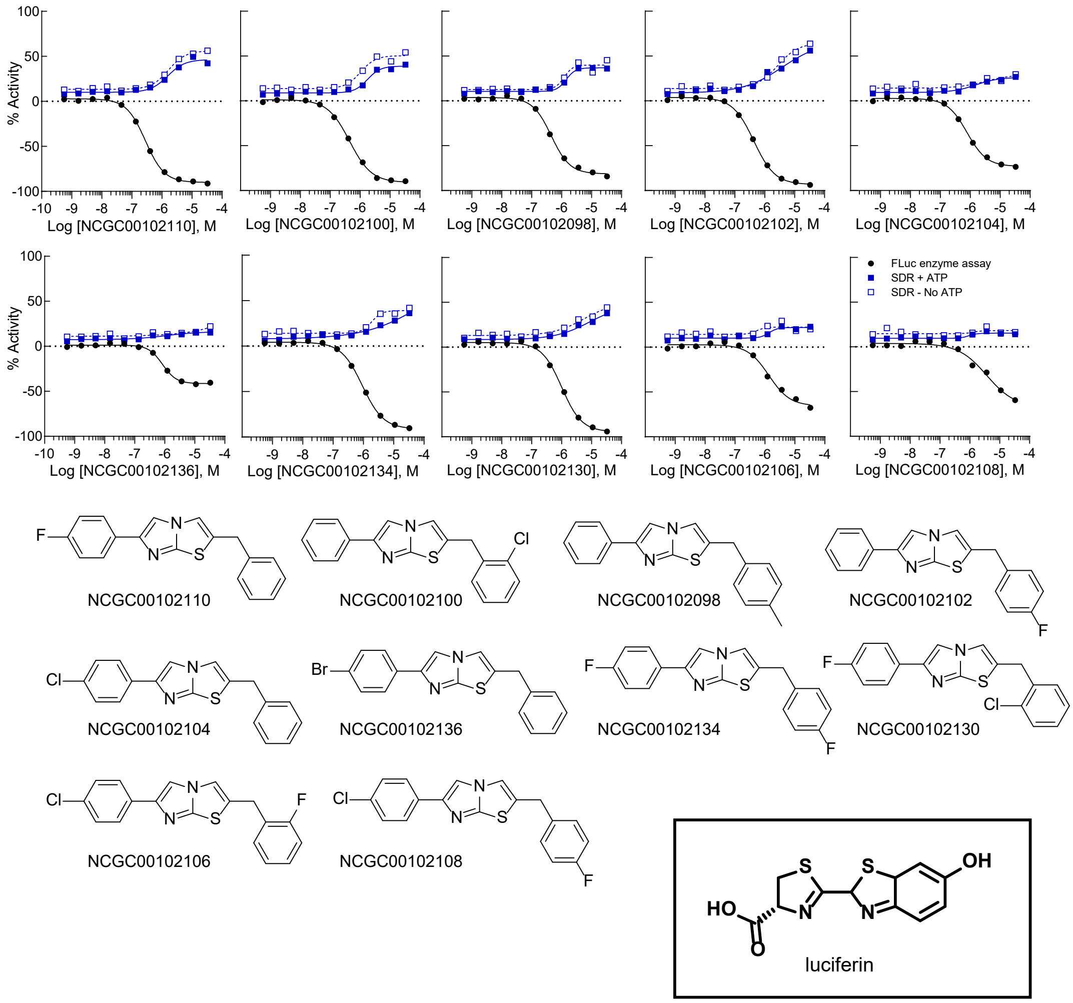


**Supplementary Figure 4. SAR correlation for enzyme and SDR assay activity from FLuc library clade O.** Concentration response curves for the imidazo[2,1-b]thiazole series (clade O), with the respective structures shown, with similarity to luciferin (box). See **Supplementary Data 2** and AIDs 1963320, 1963319 and 1963318 for source data.

**Supplementary Figure 5**. Expanded clade S evaluated for ATP dependence with SDR and functional enzyme assays

a

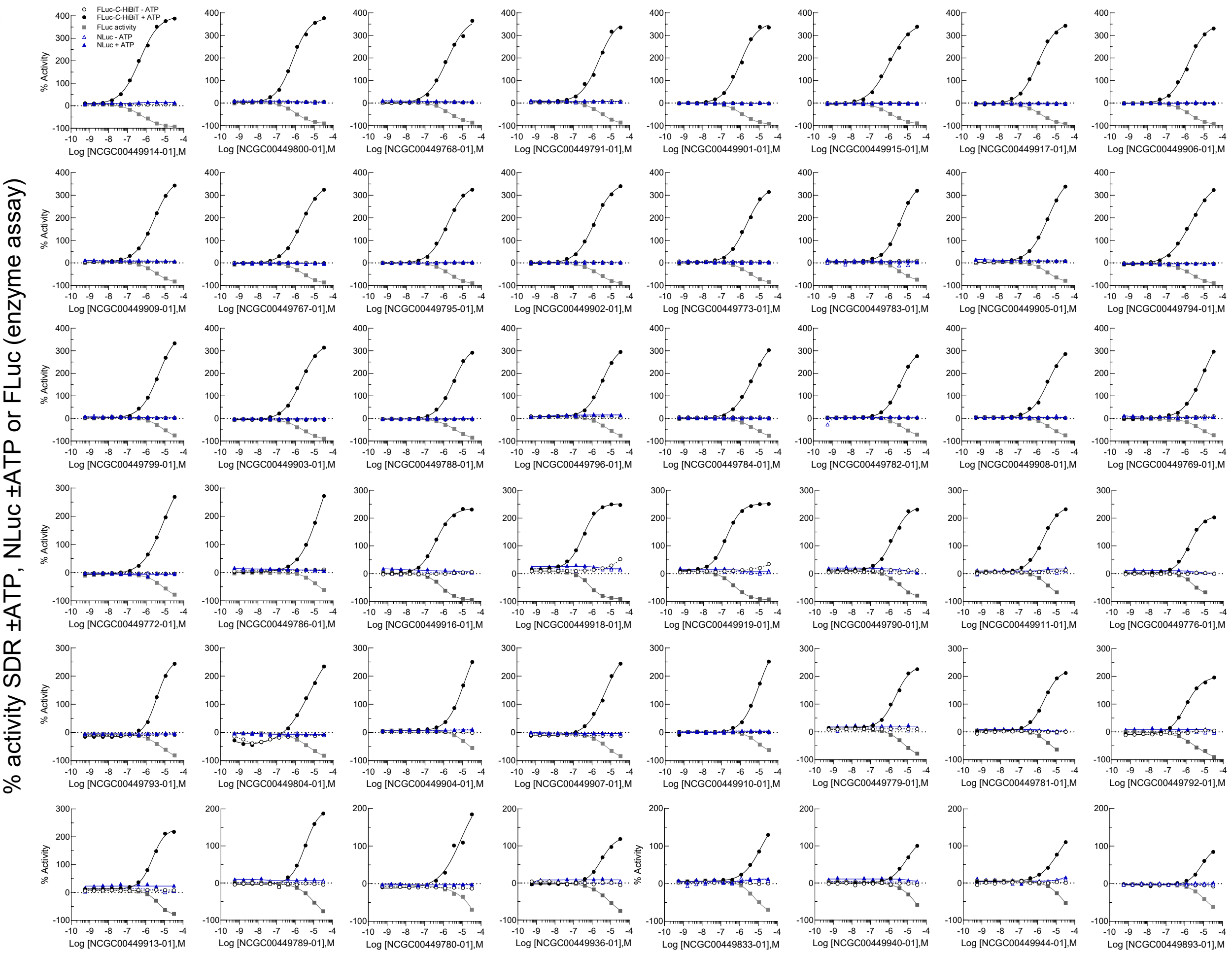


b

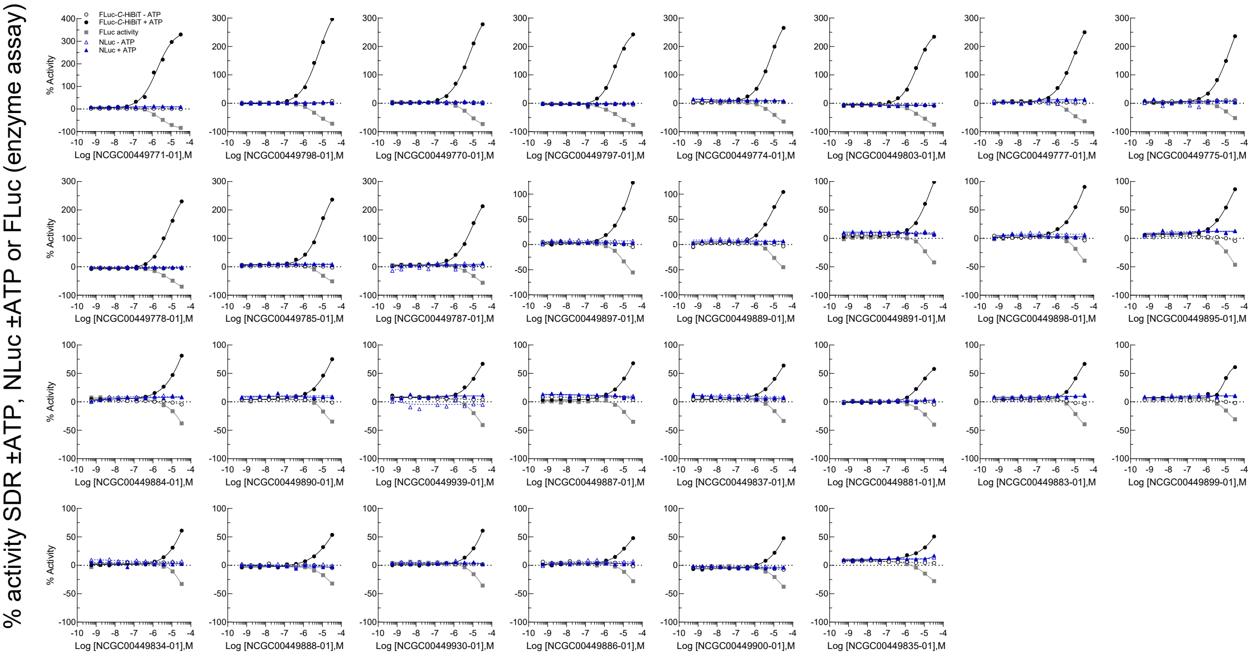

**Supplementary Figure 5**. Expanded clade S evaluated for ATP dependence with SDR and functional enzyme assays

**c**

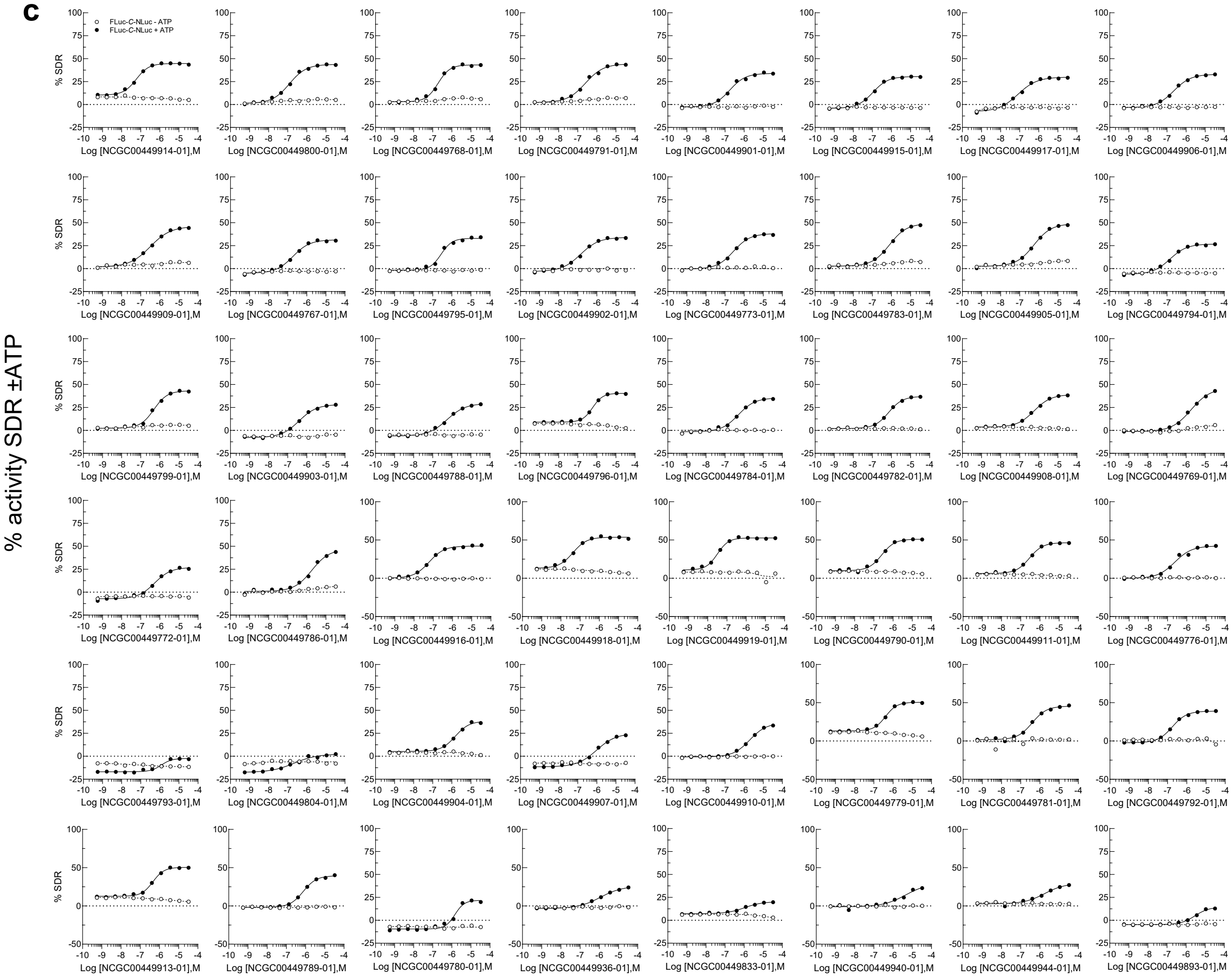


**d**

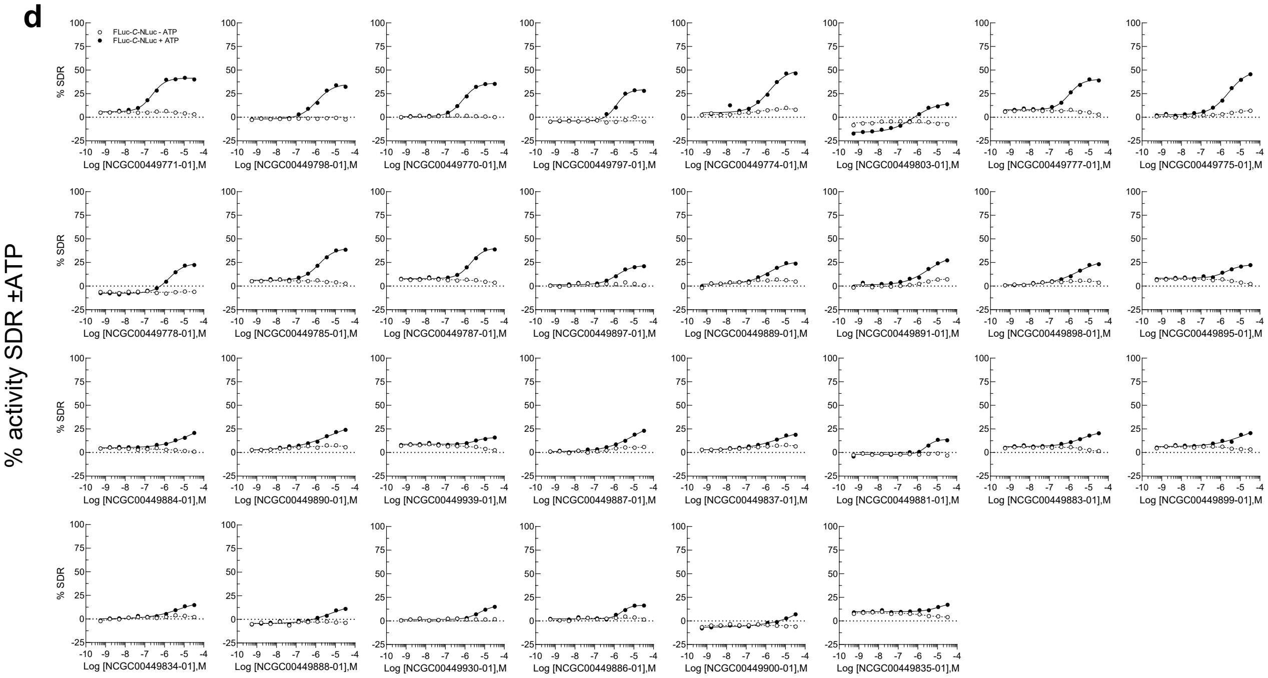

**Supplementary Figure 5**. Expanded clade S evaluated for ATP dependence with SDR and functional enzyme assays

e

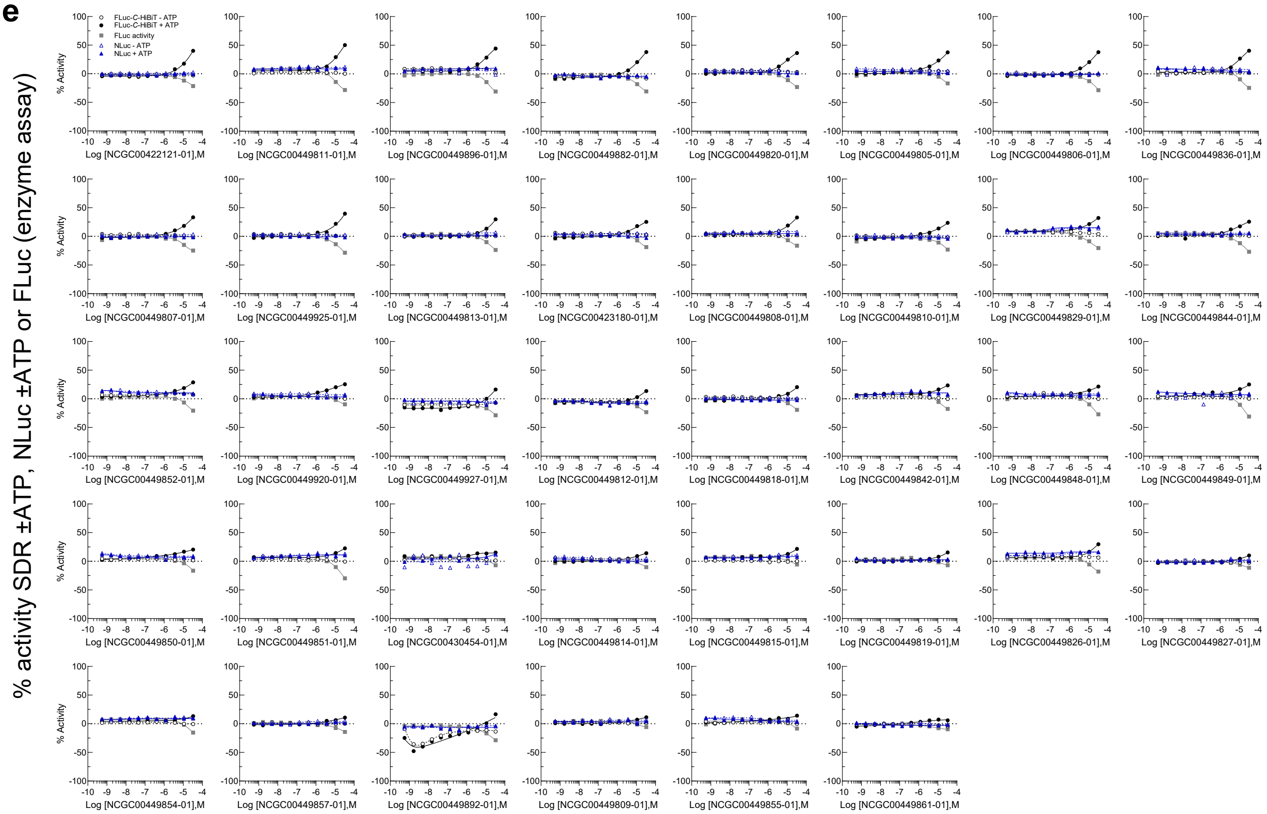


f

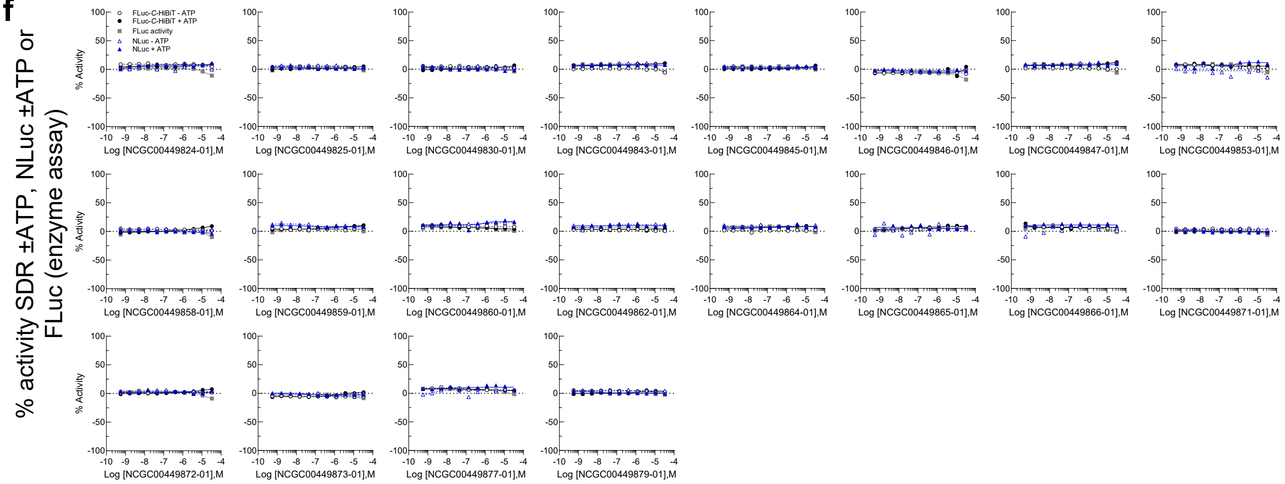

**Supplementary Figure 5**. Expanded clade S evaluated for ATP dependence with SDR and functional enzyme assays

g

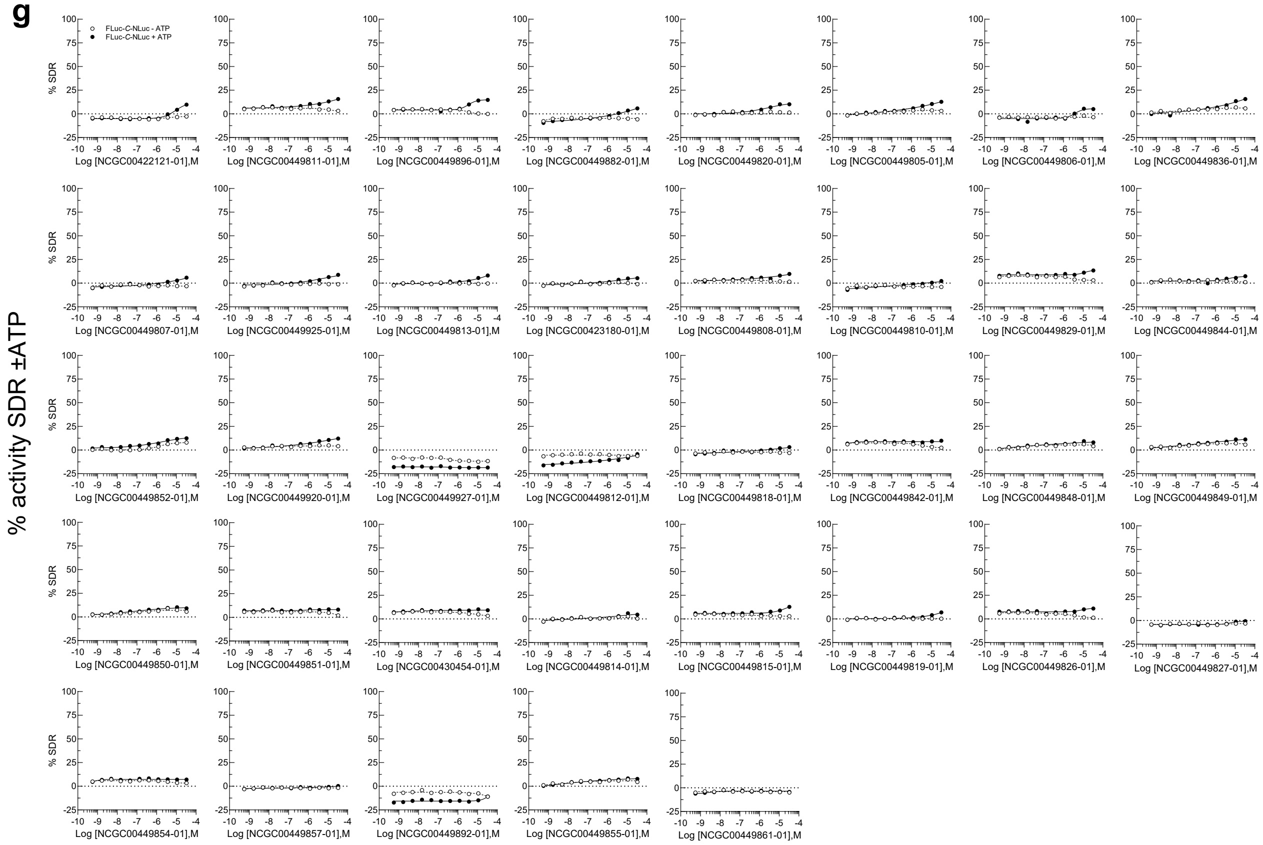


h

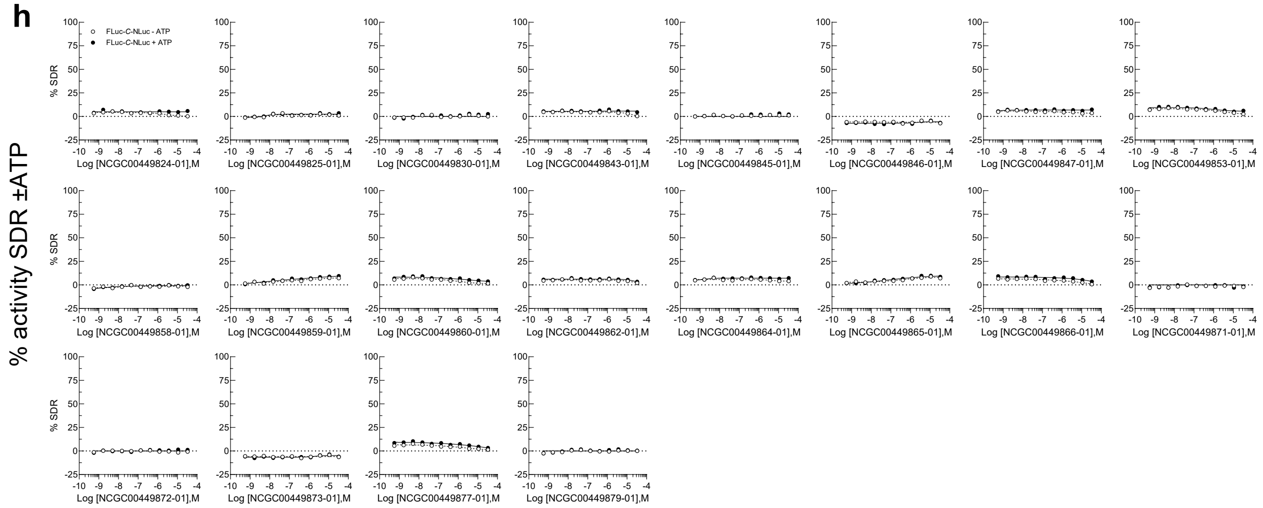


**Supplementary Figure 5**. **Expanded clade S evaluated for ATP dependence with SDR and functional enzyme assays.** Part 1, **a**, Retest of clade S compounds identified in qHTS on FLuc-*C*-HiBiT (black circles), Nluc (blue triangles) and *P. pyralis* FLuc (gray squares) and on **b**, additional clade S analogs (active). Part 2, **c**, Test of clade S compounds identified in qHTS on FLuc-*C*-NLuc and on **d**, additional clade S analogs (active). Part 3, **e**, additional clade S analogs (moderately active) and **f,** inactive on FLuc-*C*-HiBiT and *P. pyralis* FLuc. Part 4, **g**, additional clade S analogs (moderately active) and **h,** inactive on FLuc-*C*-NLuc. Assays were run in the presence (solid symbols) or absence (open symbols) of 10 μM ATP. Corresponding SMILES for NCGC IDs and source data are provided in **Supplementary Data 3** and as a Source Data file.

**Supplementary Figure 6**. Comparison of SDR and Thermofluor analysis of select clade S analogs

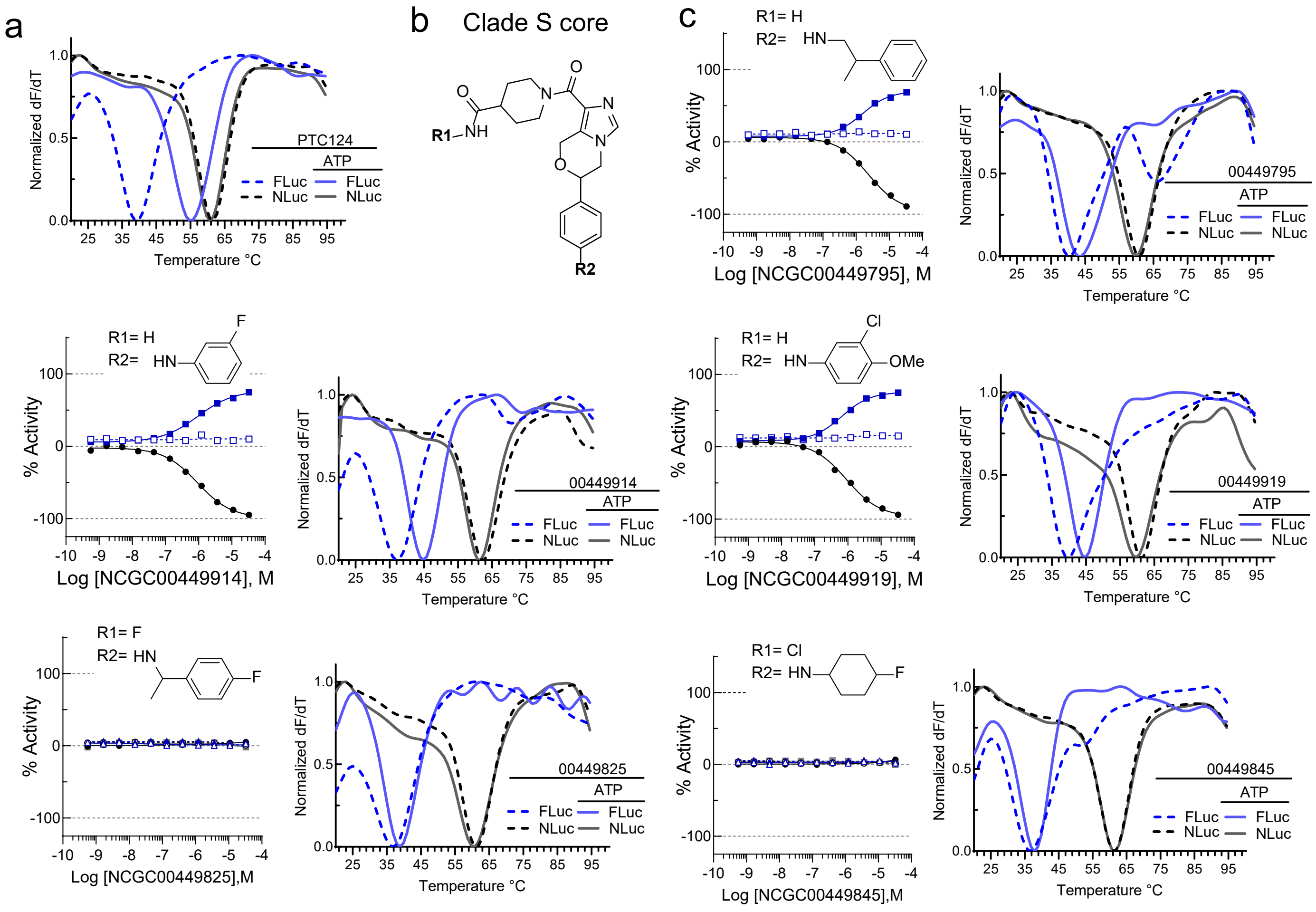


**Supplementary Figure 6. Comparison of SDR and Thermofluor analysis of select clade S analogs. a**, Thermofluor ATP-sensitive control using PTC124 (10 μM) ±ATP (2 mM). **b**, Clade S series core is shown. **c**, SDR (left panel) CRCs for clade S analogs from the FLuc-enriched compound library qHTS in the presence of 10 μM ATP (solid blue squares) or absence of ATP (open blue squares). Functional firefly luciferase (FLuc) enzyme assay (solid black circles). Corresponding Thermofluor assay (right panel), for indicated clade S analogs ±compound (100 μM) with ATP (2 mM) with either FLuc (5 μM) or NLuc (5 μM). Source data are provided as a Source Data file.

**Supplementary Figure 7**. Limit of detection of basal and ligand-mediated ABL1-*N*-HiBiT SDR output.

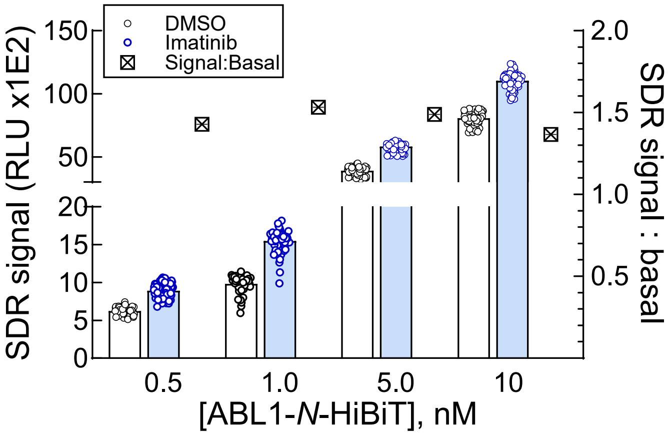


**Supplementary Figure 7. Limit of detection of basal and ligand-mediated ABL1-*N*-HiBiT SDR output.** White bars and open black circles represent DMSO basal SDR signal for apo ABL1-*N*-HiBIT plus LgBiT ω-fragment and furimazine (FMZ) substrate. Blue bars and open blue circles represent imatinib (33 μM) dependent enhanced SDR signal. ☒ represent signal-to-background for SDR assay response from addition of indicated concentration of ABL1-*N*-HiBiT. Error bars represent SD, n=96 replicate wells for all concentrations of ABL1-*N*-HiBiT except 1 nM where n=64 wells. Source data are provided as a Source Data file.

**Supplementary Figure 8**. Heatmap displaying ABL1 and PKA $pIC_{50}$ or $pSDR_{50}$ for 128 kinase inhibitors

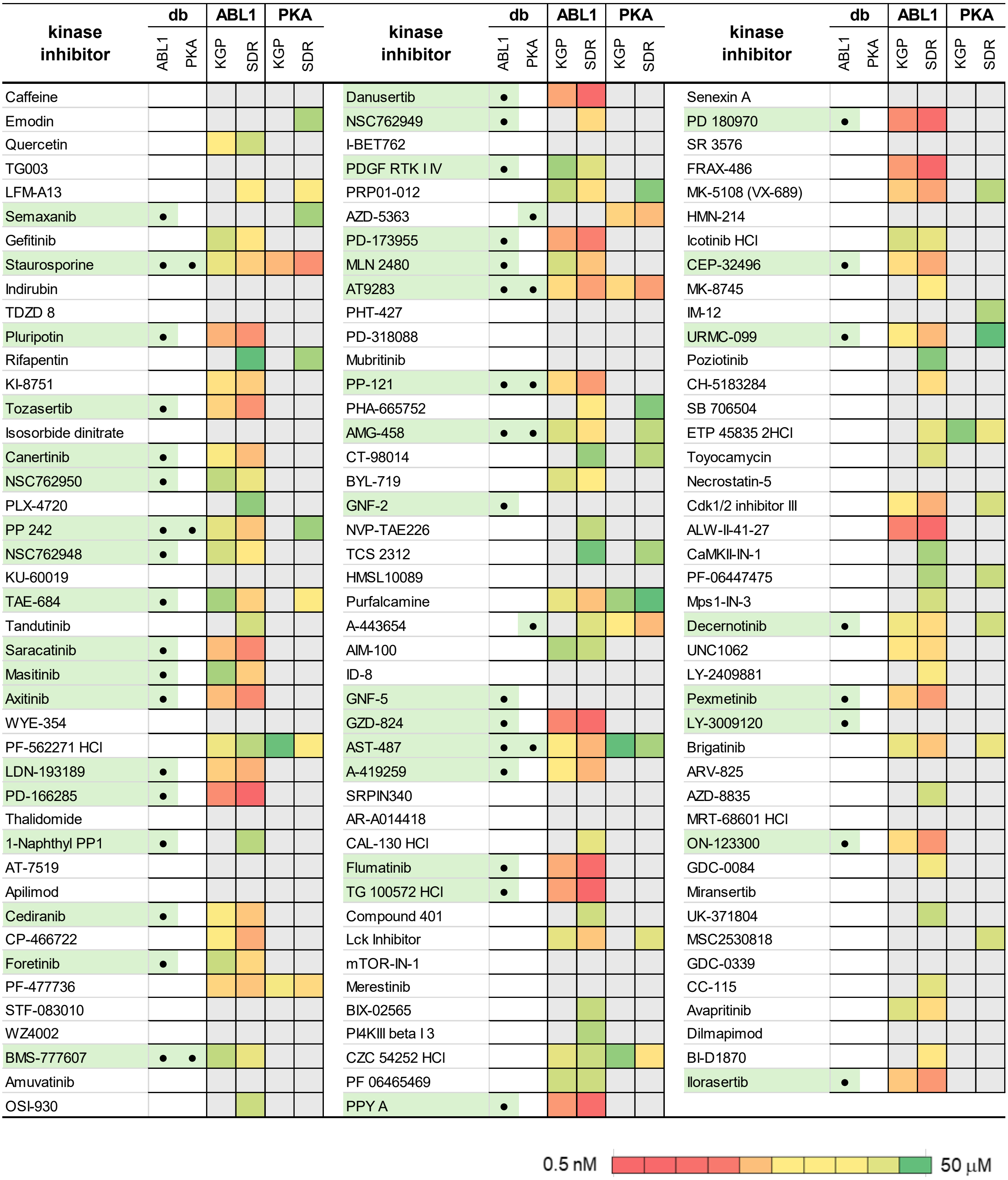


**Supplementary Figure 8**. Heatmap displaying ABL1 and PKA $pIC_{50}$ or $pSDR_{50}$ for 128 kinase inhibitors. Source data are provided as a Source Data file.

**Supplementary Figure 9.** ABL1-*N*-HiBiT enzyme inhibition and SDR assay activity (part 1)

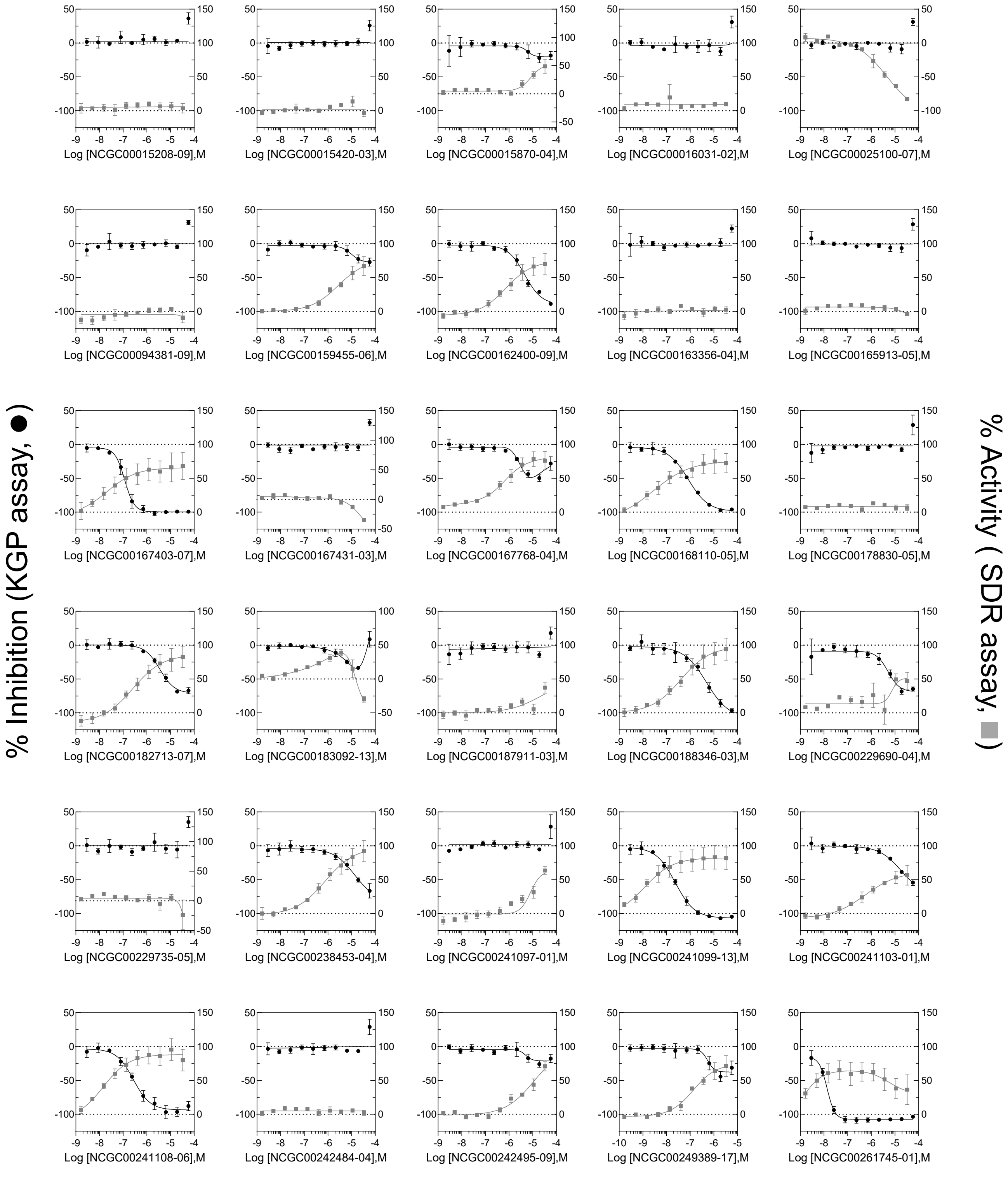

**Supplementary Figure 9.** ABL1-*N*-HiBiT enzyme inhibition and SDR assay activity (part 2)

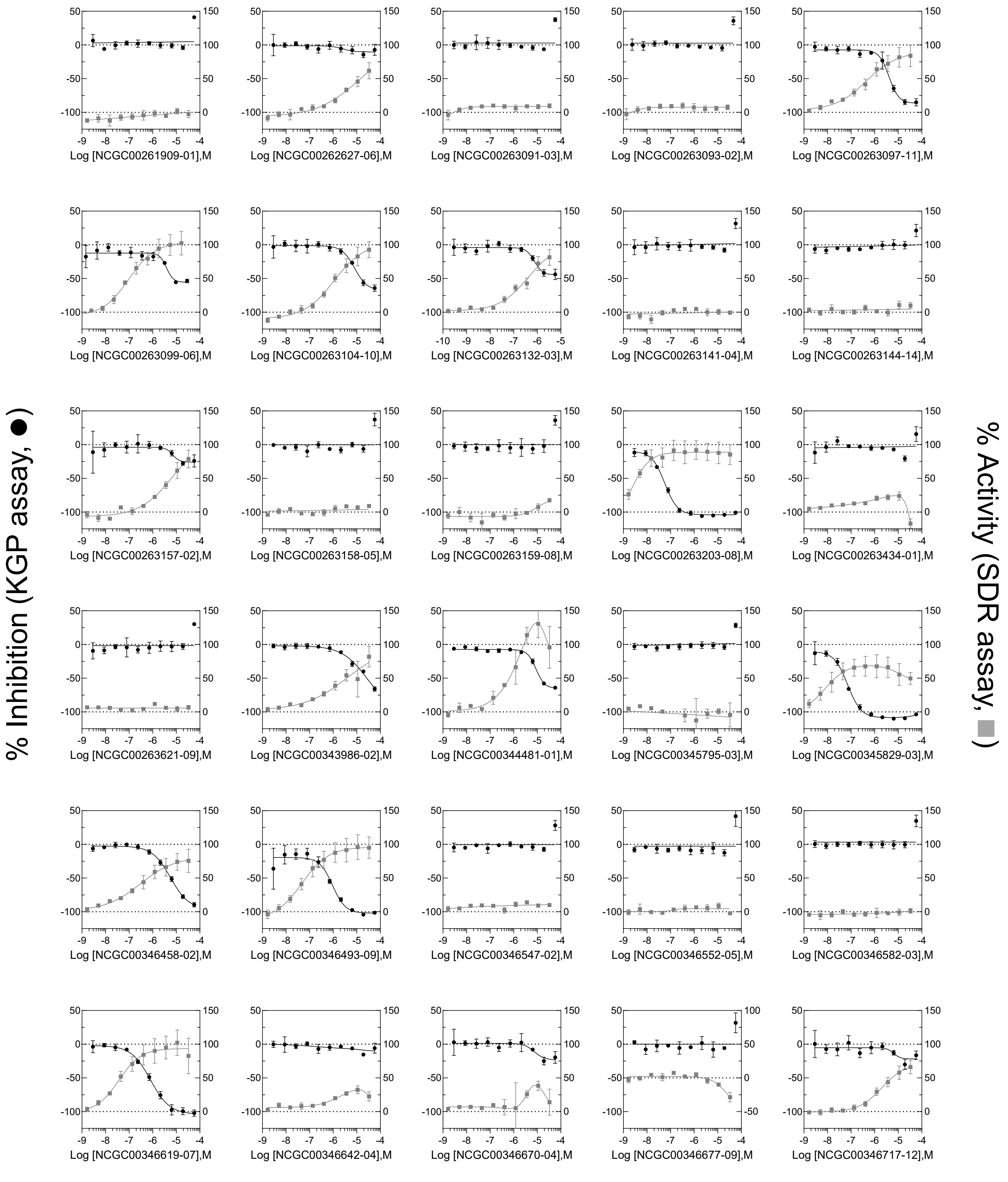

**Supplementary Figure 9.** ABL1-*N*-HiBiT enzyme inhibition and SDR assay activity (part 3)

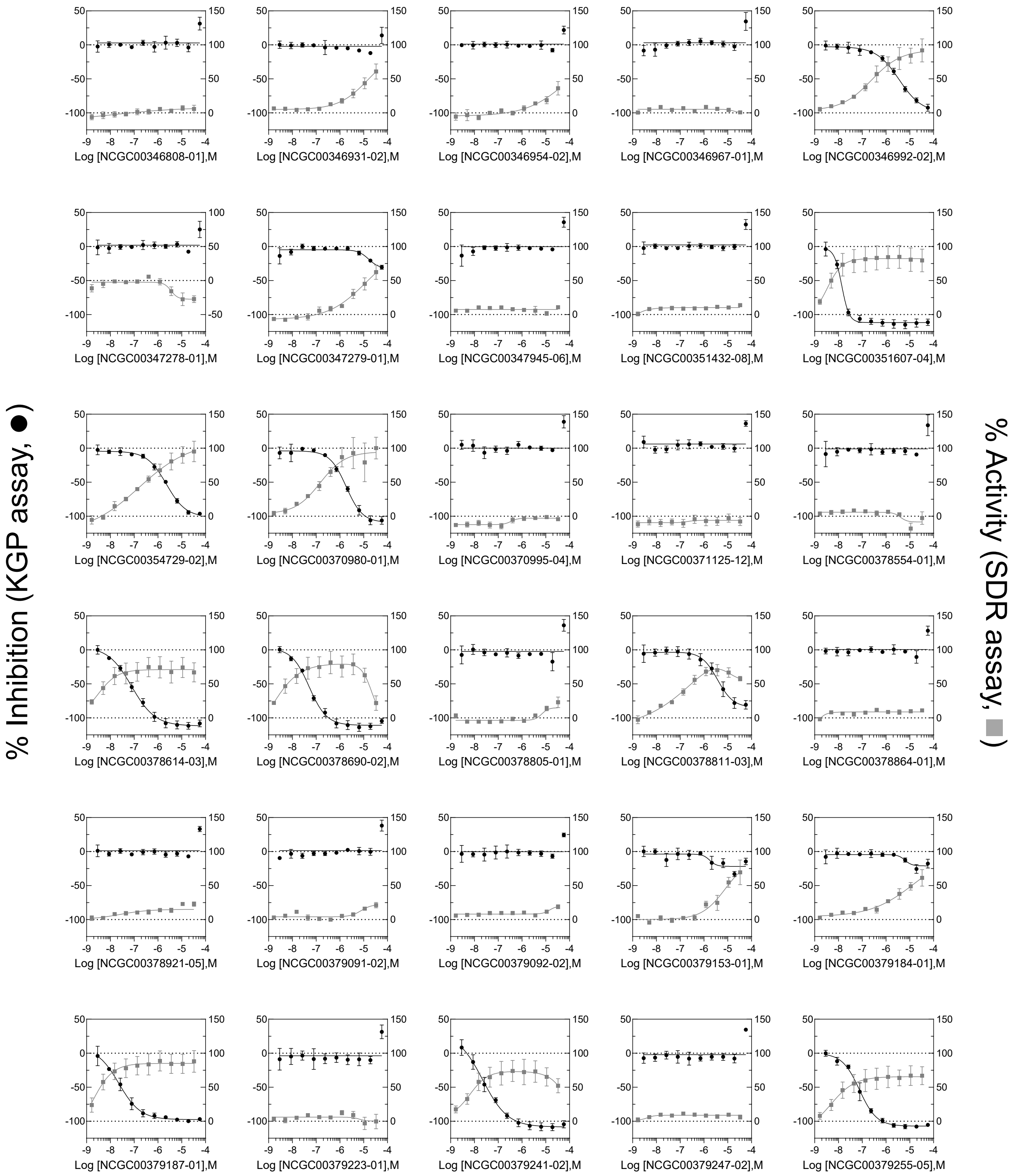

**Supplementary Figure 9.** ABL1-*N*-HiBiT enzyme inhibition and SDR assay activity (part 4)

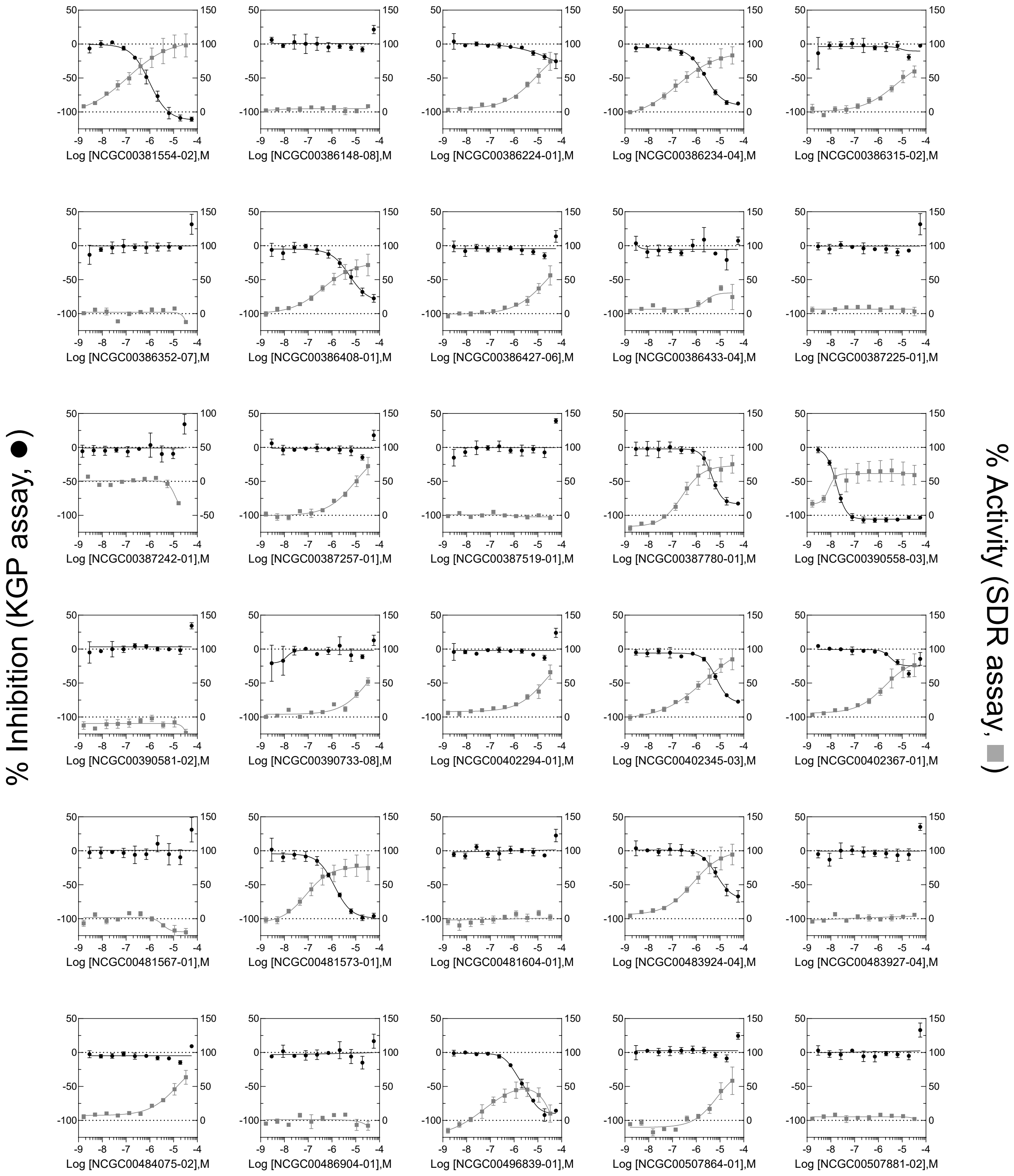

**Supplementary Figure 9.** ABL1-*N*-HiBiT enzyme inhibition and SDR assay activity (part 5)

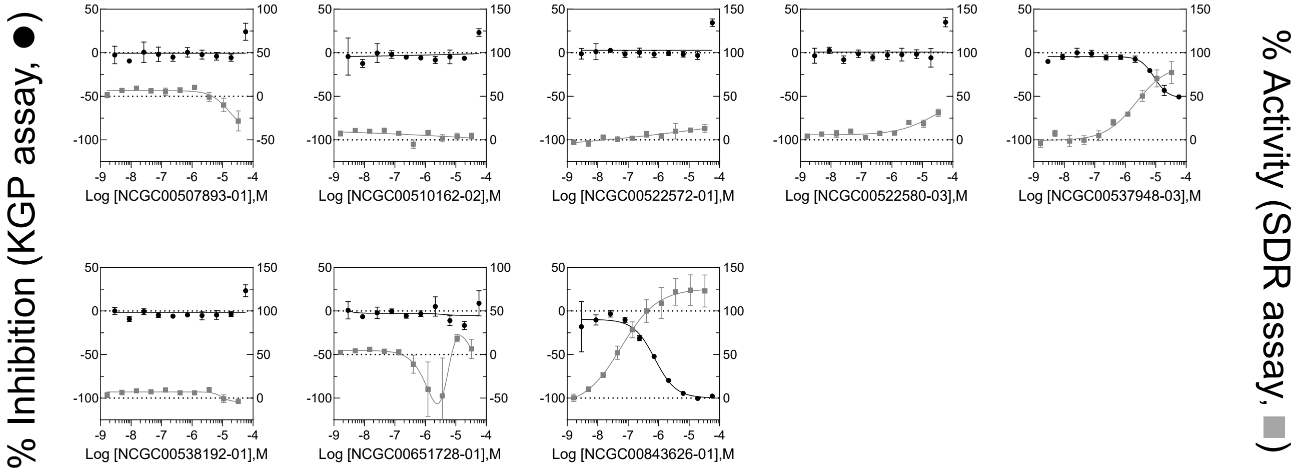


**Supplementary Figure 9. ABL1-*N*-HiBiT enzyme inhibition and SDR assay activity from a 128-member kinase inhibitor library**. The functional enzyme KGP assay (solid black circles) is shown on the left axis and the SDR assay (solid gray squares) on the right axis. Error bars represent SD, n=3 technical replicates. The lowest concentration point for each compound for all assays was removed due to a dispense error. NCGC IDs corresponding to compound names and SMILES provided in **Supplementary Data 4**, and source data are provided in **Supplementary Data 5** and as a Source Data file. Full data available in AIDs 1963317 and 1963315.

**Supplementary Figure 10.** Allosteric ligand effect on ABL1-*N*-HiBiT enzyme activity

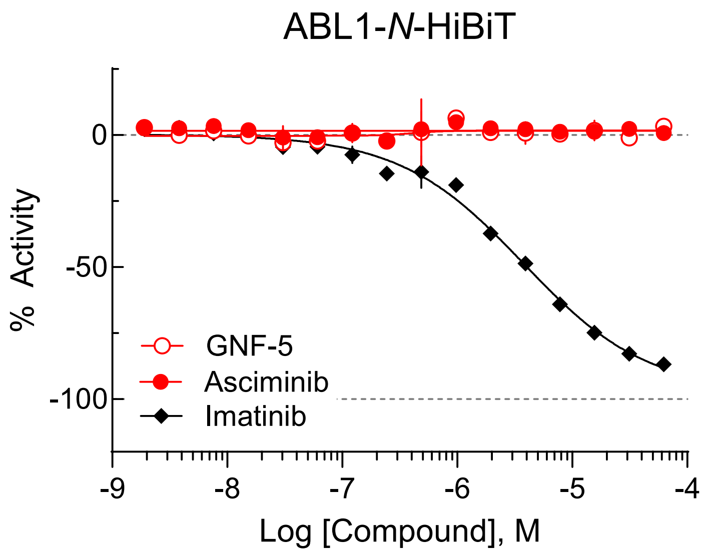


**Supplementary Figure 10. Allosteric ligand effect on ABL1-*N*-HiBiT enzyme activity.** Enzyme activity was determined with the KGP assay and data was normalized to no enzyme as 100% inhibition. Error bars are SEM, n=2 technical replicates. Source data are provided as a Source Data file.

**Supplementary Figure 11**. PKA-*N*-HiBiT enzyme inhibition and SDR assay activity (part 1)

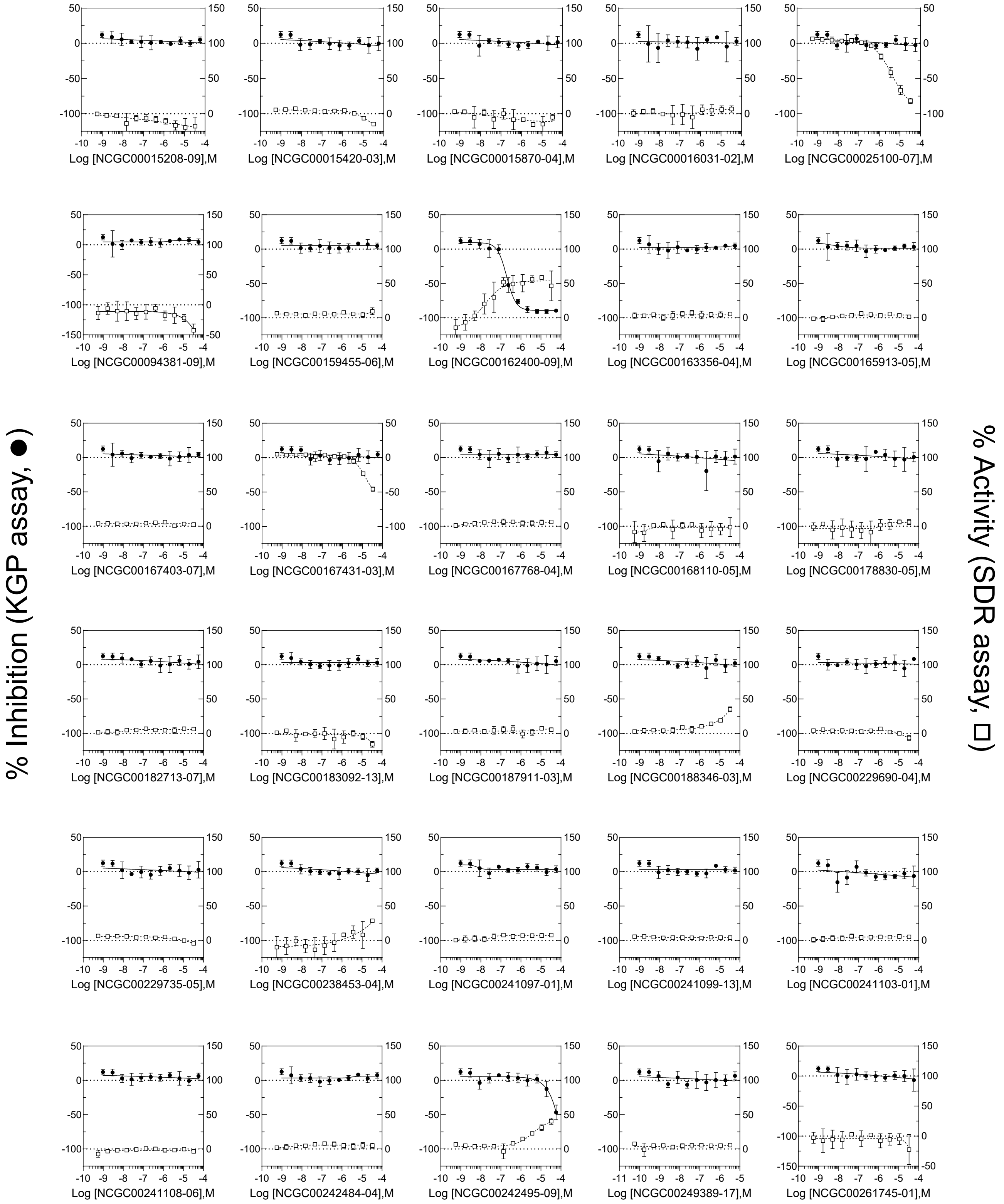

**Supplementary Figure 11**. PKA-*N*-HiBiT enzyme inhibition and SDR assay activity (part 2)

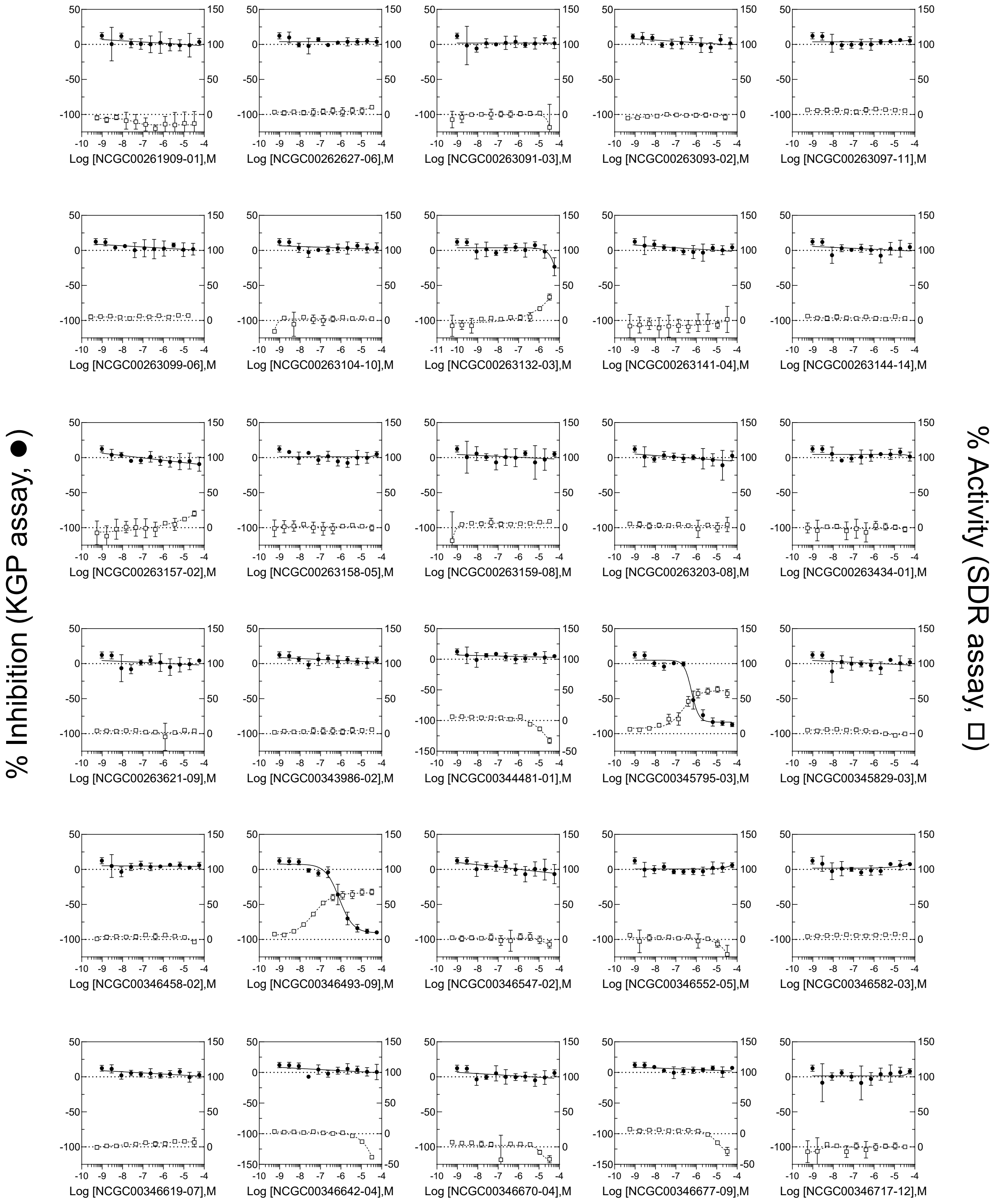

**Supplementary Figure 11**. PKA-*N*-HiBiT enzyme inhibition and SDR assay activity (part 3)

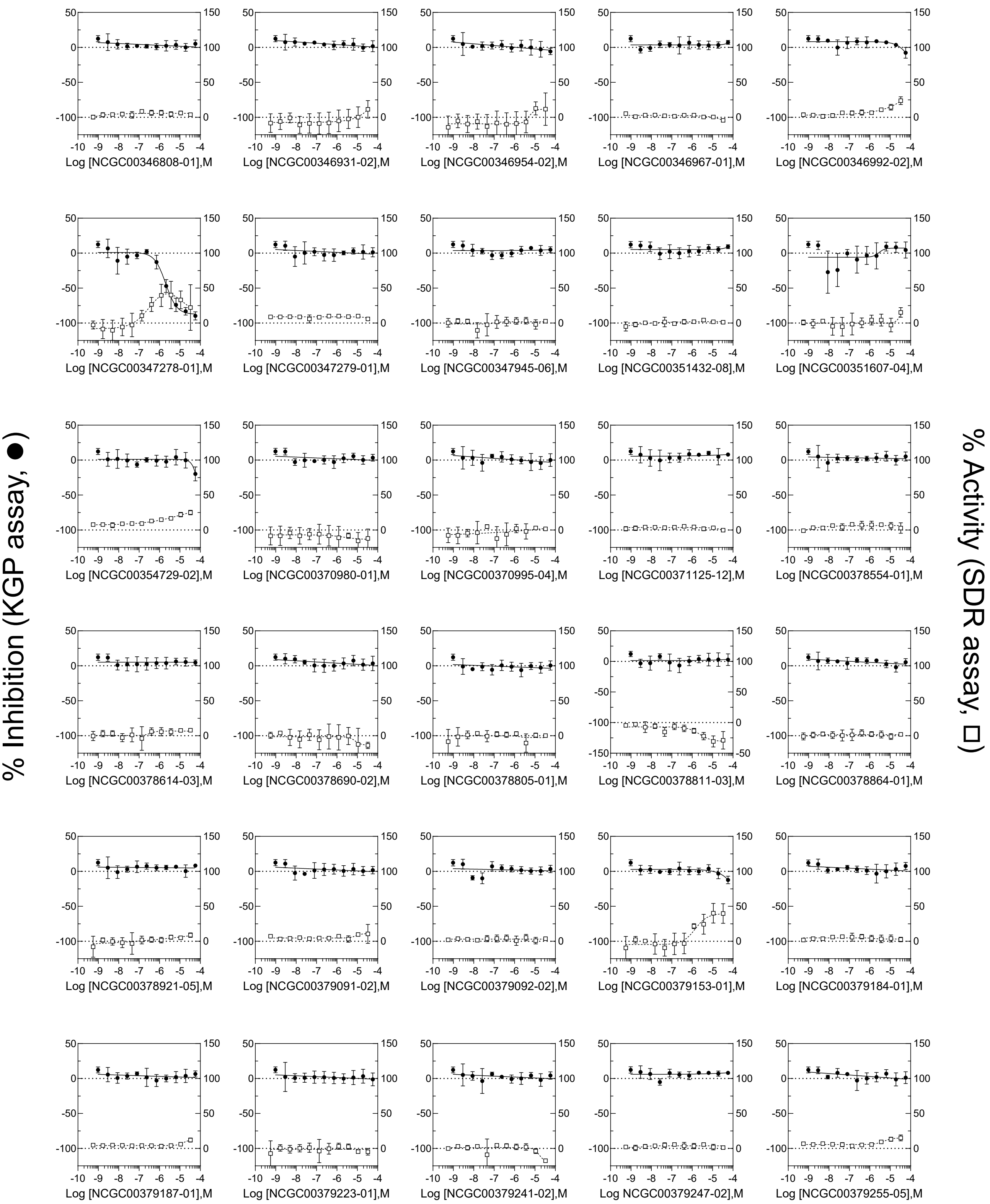

**Supplementary Figure 11**. PKA-*N*-HiBiT enzyme inhibition and SDR assay activity (part 4)

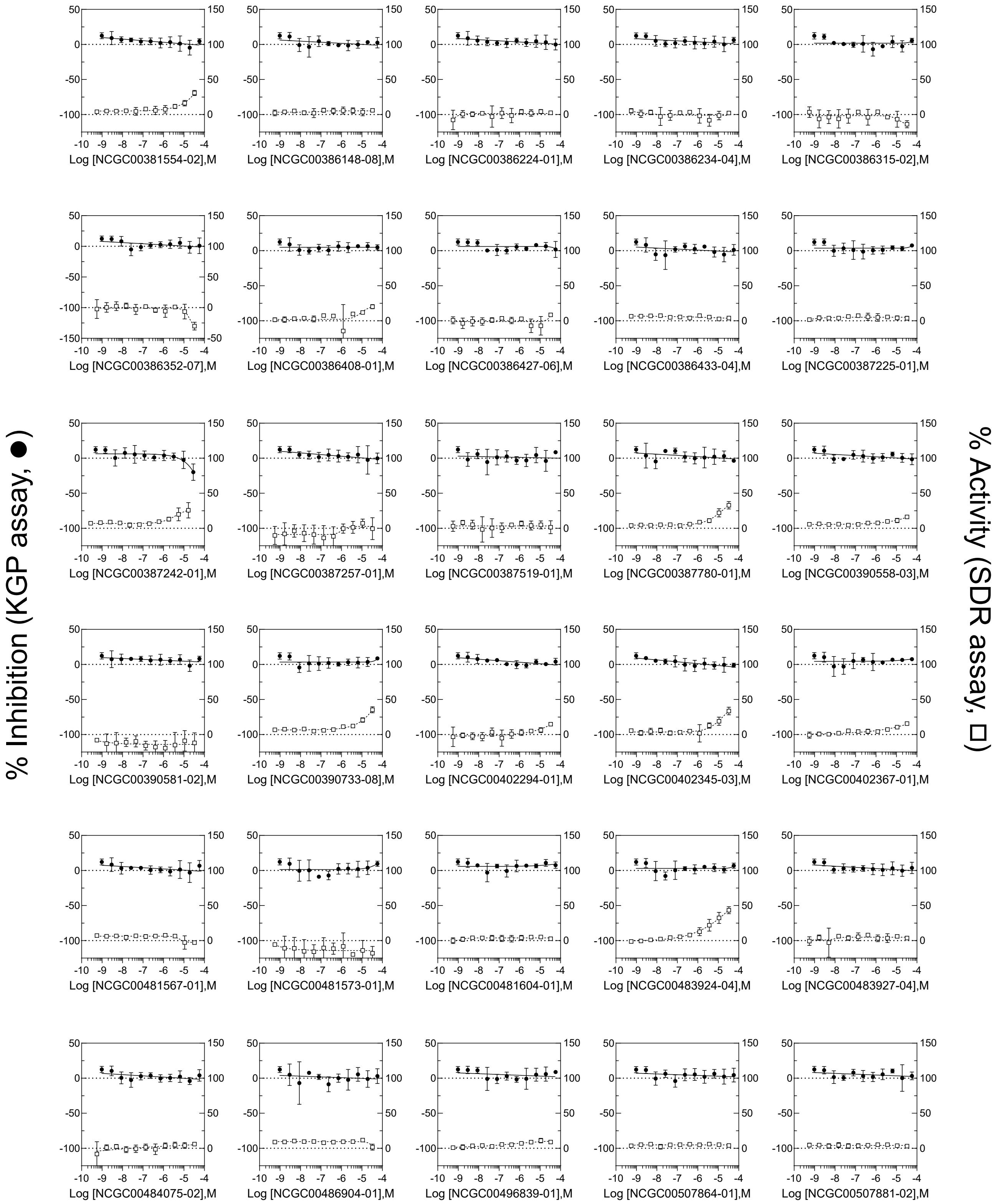

**Supplementary Figure 11**. PKA-*N*-HiBiT enzyme inhibition and SDR assay activity (part 5)

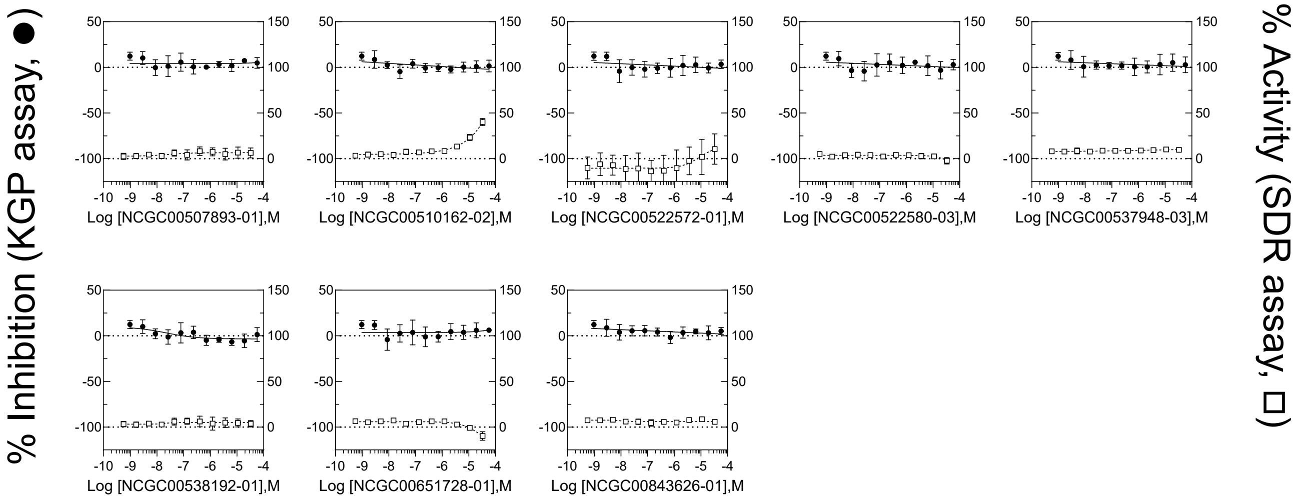


**Supplementary Figure 11. PKA-*N*-HiBiT enzyme inhibition and SDR assay activity from a 128-member kinase inhibitor library**. The functional enzyme KGP assay (solid circles) is shown on the left axis and the SDR assay (open square) on the right axis. Error bars represent SD, n=3 technical replicates. NCGC IDs corresponding to compound names and SMILES provided in **Supplementary Data 4**, and source data are provided in **Supplementary Data 6** and as a Source Data file. For full data see AIDs 1963321 and 1963322.

**Supplementary Figure 12**. Limit of detection of basal and ligand-mediated iPGM-*C*-HiBiT SDR output.

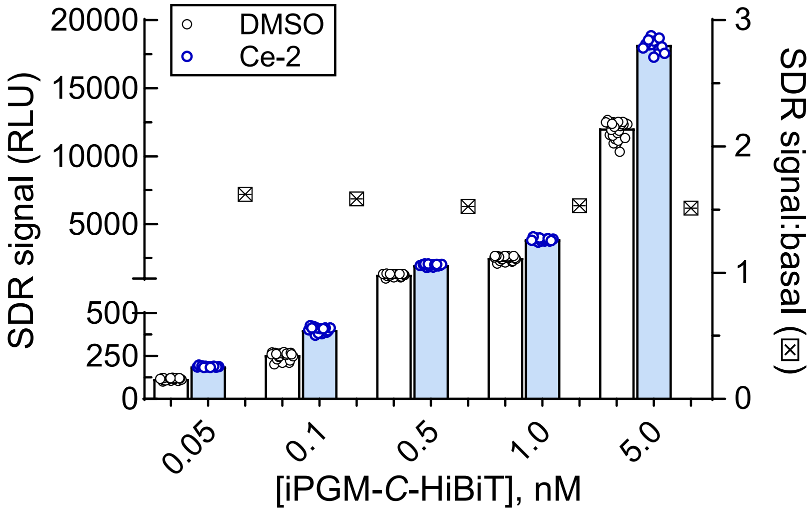


**Supplementary Figure 12. Limit of detection of basal and ligand-mediated iPGM-*C*-HiBiT SDR output.** White bars and open black circles represent DMSO basal SDR signal for apo iPGM-*C*-HiBiT plus LgBiT ω-fragment and furimazine (FMZ) substrate. Blue bars and open blue circles represent ipglycermide Ce-2 (1 μM) dependent enhanced SDR signal. ⊠ represent signal-to-background for SDR assay response from addition of indicated concentration of iPGM-*C*-HiBiT. Error bars represent SD, n=32 (DMSO) and n=14 (Ce-2) replicate wells. Source data are provided as a Source Data file.

**Supplementary Figure 13.** Estimation of DHFR-*C*-HiBiT concentration from cellular lysate

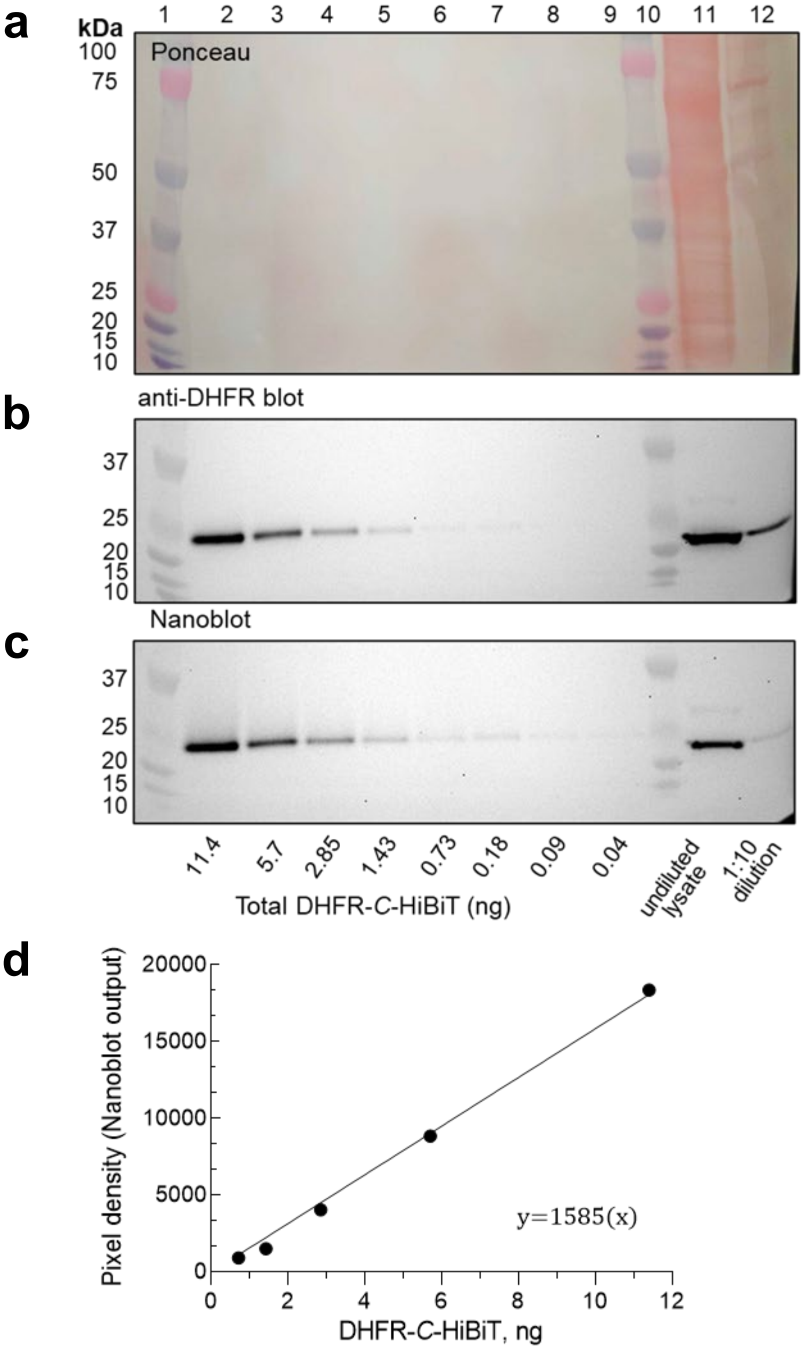


**Supplementary Figure 13. Estimation of DHFR-*C*-HiBiT concentration from cellular lysate**. **a**, Ponceau stained nitrocellulose membrane from transfer of purified recombinant DHFR-*C*-HiBiT expressed in *E. coli* (lanes 2-9) and HEK293 cellular lysate, undiluted and 1:10 dilution, from gene edited cells installing *C*-terminal HiBiT sequence on DHFR, lanes 11 and 12, respectively. **b**, Anti-DHFR blot, anti-DHFR (Abcam, Cat # ab124814; Clone# EPR5285; RRID:AB_10975115) 1:1000 o/n 4°C, anti-rabbit IgG HRP (Invitrogen, Cat # A16096; polyclonal secondary; RRID:AB_2534770) 1:1000 2h RT. **c**, Nanoblot obtained using luminescence detection from reconstituted NLuc after incubation with LgBiT o/n at 4°C using detection reagent (Promega, Cat # N2410). **d**, Linear regression from densitometry analysis of the Nanoblot for the estimation of cellular lysate DHFR-*C*-HiBiT concentration. Quantities of recombinant DHFR-*C*-HiBiT (ng) and dilutions of cell lysate shown at bottom of Nanoblot. Blots are a representative of 3 replicates. Source data are provided as a Source Data file and uncropped blots are shown at the end of Supplementary Information.

**Supplementary Figure 14.** Basal RLU range from HiBiT tagged proteins

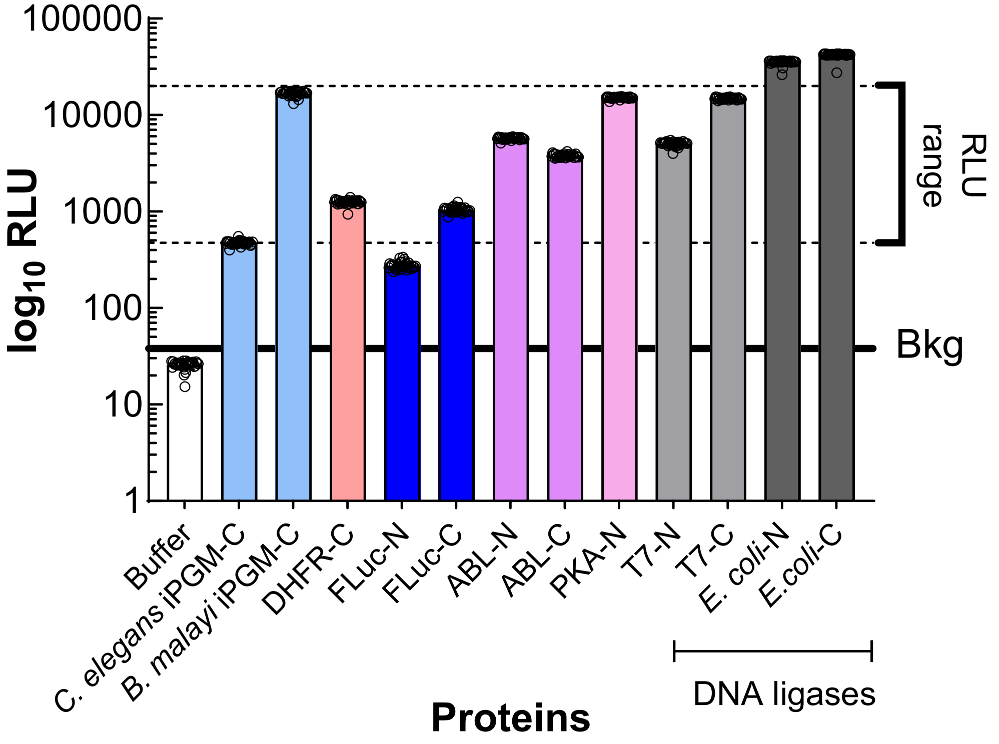


**Supplementary Figure 14. Basal RLU range from HiBiT tagged proteins.** Luminescence output are from 10 nM protein using the SDR assay described in **Methods**. The position of the HiBiT tag is indicated as *N*- or *C*-terminal fusion. White bar indicates buffer control without a HiBiT tagged protein, used to indicated the background reader luminescence. Error bars represent SD, n=32 replicate wells. One data point was removed from FLuc-*N* due to dispense error. Source data are provided as a Source Data file.

**Supplementary Figure 15**. Correlation between FLuc-*C*-HiBiT SDR % activity (max. response) vs. $pSDR_{50}$ across select compounds sets.

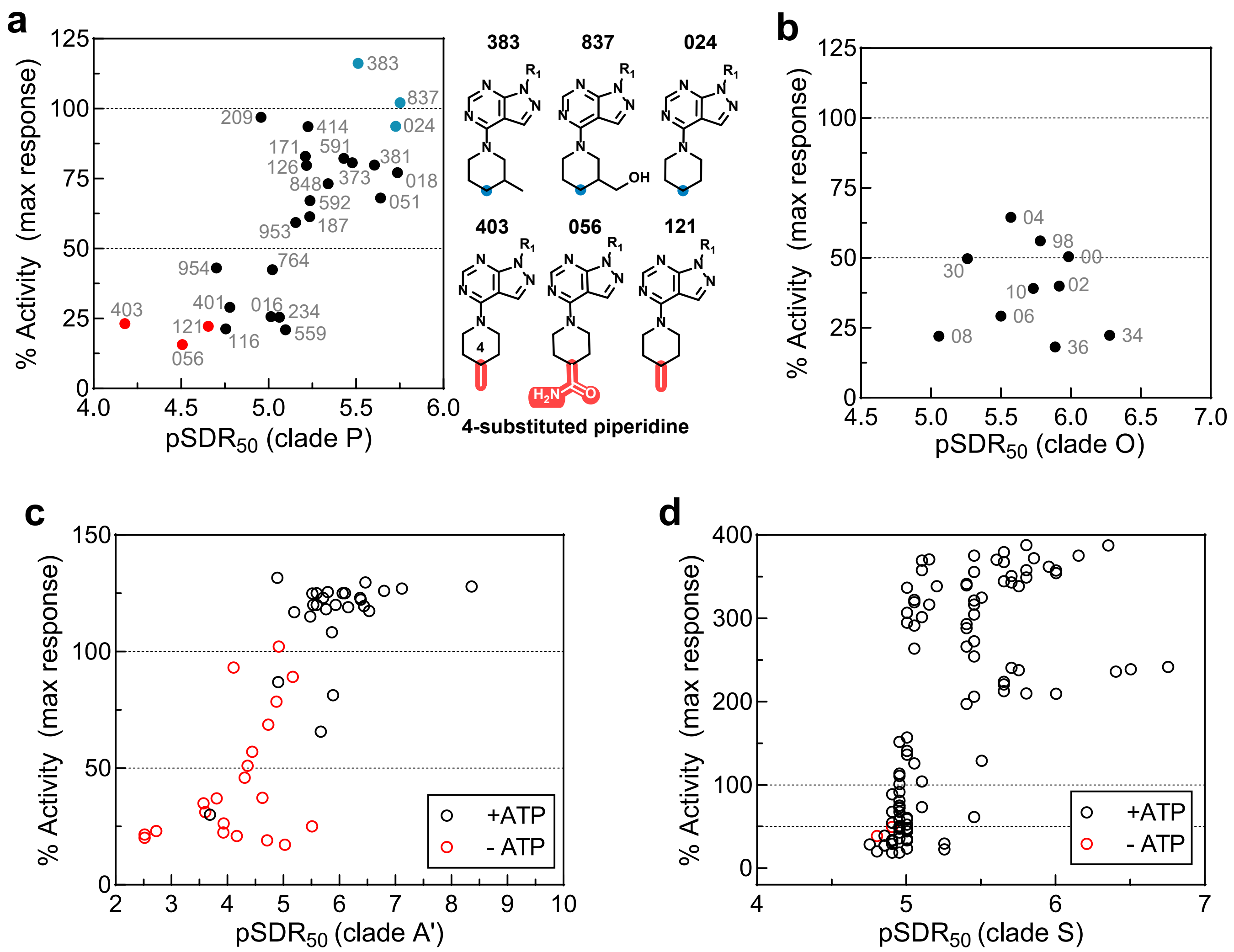


**Supplementary Figure 15. Correlation between FLuc-*C*-HiBiT SDR % activity (max. response) vs. $pSDR_{50}$ across select compounds sets**. % Activity vs $pSDR_{50}$ for the ATP-independent **a**, pyrazolo pyrimidine (clade P – with example analogs), and **b** imidazo[2,1-b]thiazole (clade O) series. Last three or two numbers of NCGC ID are used to ID data points in **a** and **b**, respectively. **c** and **d,** Relationship between SDR % activity (maximum (max) response) and $pSDR_{50}$ for the substituted indoline (clade A') and dihydro-imidazo-oxazine (clade S), respectively, for the ±ATP condition. Data from **Supplementary Data 2** (clade A') and **Supplementary Data 3** (expanded clade S). Source data are provided as a Source Data file.

**Supplementary Figure 16**. SDS-PAGE analysis of recombinant *E. coli* expressed and purified proteins.

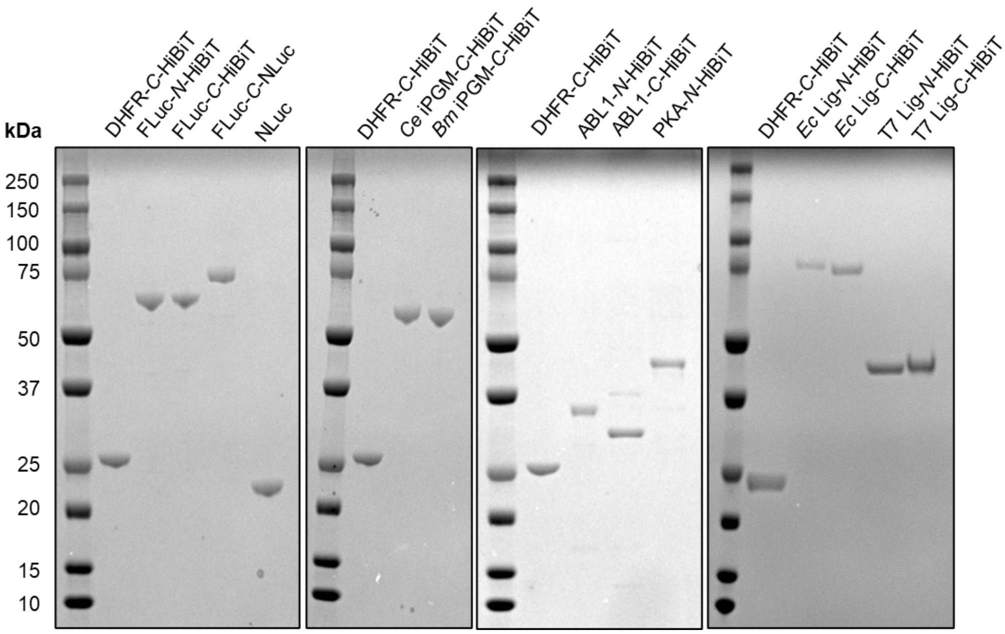


**Supplementary Figure 16. SDS-PAGE analysis of recombinant *E. coli* expressed and purified proteins**. The relative purity of each purified protein-*N/C*-HiBiT or -*C*-NLuc construct was assessed by denaturing SDS PAGE stained with Coomassie Blue R-250 and destained for 2.5 h. Total protein concentration was first measured by BCA assay in triplicate and preliminary gel samples were prepared such that 5 µg of total protein material would be loaded in 20 µL sample volume. These samples were resolved over 4-20% denaturing gel at 180 V for 35 minutes. The relative intensity of each protein was measured by pixel densitometry using ImageJ software, and protein load was adjusted using DHFR-*C*-HiBiT as the standard of normalization. Here, each protein was loaded to have an intensity roughly equal to DHFR-*C*-HiBiT where protein purity was determined using pixel densitometry. Most proteins were >95% pure, while three showed some impurities, but were still highly pure: *B. malayi* iPGM-*C*-HiBiT, >85% pure; ABL-*N*-HiBiT, >80% pure; FLuc-*N*-HiBiT, >90% pure. *Ce*, *C. elegans*; *Bm*, *B. malayi*; *Ec*, *E. coli*. Uncropped gels are shown at the end of Supplementary Information.

**Supplementary Table 1**. DNA ligase ligand binding parameters.

**Supplementary Table 1**: DNA ligase ligand binding parameters

| DNA Ligase | 22-mer dsDNA | | | | | ATP | | | | | NAD+ | | | | |
|---|---|---|---|---|---|---|---|---|---|---|---|---|---|---|---|
| | $SDR_{50}$, M | SD | max resp | SD | n | $SDR_{50}$, M | SD | max resp | SD | n | $SDR_{50}$, M | SD | max resp | SD | n |
| *E. coli* Lig-*N*-HiBiT | 2.145E-08 | 4.1E-09 | 167.6 | 24.4 | 3 | ≥1.0E-04 | NA | 48.3 | 3.5 | 3 | ≥1.0E-04 | NA | 90.7 | 4.7 | 3 |
| *E. coli* Lig-*C*-HiBiT | 6.2E-04 | 1.1E-04 | -50.6 | 1.4 | 3 | NA | NA | 7.8 | 2.9 | 3 | NA | NA | 4.9 | 0.5 | 3 |
| T7 Lig-*N*-HiBiT | 1.289E-06 | 2.2E-06 | -34.1 | 17.4 | 3 | ≥1.0E-04 | NA | 407.3 | 28.5 | 3 | NA | NA | 9.8 | 1.7 | 3 |
| T7 Lig-*C*-HiBiT | 3.624E-09 | 2.0E-09 | 115.1 | 32.5 | 3 | ≥1.0E-04 | NA | 215.2 | 5.1 | 3 | NA | NA | 8.9 | 2.3 | 3 |

Lig., ligase; SD, standard deviation; max resp, maximum response; n, number of technical replicates; NA, not applicable as there was no effect. Source data are provided as a Source Data file.

## **Supplementary Table 2**. PubChem Accession AIDs

**Supplementary Table 2**: PubChem Accession AIDs

| Target protein | PubChem assay name | Assay format | Output type | PubChem AID |
|---|---|---|---|---|
| Firefly luciferase (oxidoreductase) | Firefly luciferase (FLuc-*C*-HiBiT) enzymatic inhibition screen - Functional assay | enzyme activity | Inhibition | 1963320 |
| | Firefly luciferase (FLuc-*C*-HiBiT) SDR gain of signal screen - plus ATP | SDR (+cofactor) | Activation | 1963319 |
| | Firefly luciferase (FLuc-*C*-HiBiT) SDR gain of signal screen - without ATP | SDR (–cofactor) | Activation | 1963318 |
| Abelson kinase (tyrosine protein kinase) | ABL1 kinase (ABL1-*N*-HiBiT) enzymatic inhibition screen - Functional assay | enzyme activity | Inhibition | 1963317 |
| | ABL1 kinase (ABL1-*N*-HiBiT) SDR gain of signal screen | SDR | Activation | 1963315 |
| Protein kinase A (serine/threonine protein kinase) | PKA kinase (PKA-*N*-HiBiT) enzymatic inhibition screen - Functional assay | enzyme activity | Inhibition | 1963321 |
| | PKA kinase (PKA-*N*-HiBiT) SDR gain of signal screen | SDR | Activation | 1963322 |

## **Supplementary Table 3**. Protocol for Thermofluor assay

**Supplementary Table 3:** Protocol for Thermofluor assay

| Sequence | Parameter | Value | Description |
|---|---|---|---|
| 1 | Enzyme | 5 µM | No enzyme control and enzyme solutions in PBS Buffer; MicroAmp Optical 384-well Reaction Plate (Applied Biosystems by Life Technologies) |
| 2 | Compound | 100 µM | Compounds in PBS added to the enzyme (100 µM) |
| 3 | Incubation | 10 mins | Room temperature, protected from light |
| 4 | Dye | 10X | Sypro Orange dye in PBS added to the sample |
| 5 | Centrifugation | 2 min | Centrifuge for 2 min at 1500 RPM at RT |
| 6 | Read | ViiA7 | Read the thermal unfolding of the protein with ViiA7 real-time PCR system |

| Step | Notes |
|---|---|
| 1 | 10X PBS Buffer (without $CaCl_2$ and $MgCl_2$): 100 mM $Na_2HPO_4$ (pH 7.4), 18 mM $KH_2PO_4$, 1.37 M NaCl, 27 mM KCl in $ddH_2O$ |
| 4 | Prepare 50X dye in PBS: 1 µL Sypro Orange 5000X (Life Technologies), 99 µL $ddH_2O$. Add 4 µL to each well |
| 6 | Read the melting temperature from 25 to 95 °C using ViiA 7 real time PCR instrument by following the below instructions<br>Perform thermal denaturation in 384-well assay plate<br>(i) Place the assay plate into the Applied Biosystems ViiA7 real-time PCR instrument and open the ViiA7 RUO software. Under Experimental Properties, select the following parameters: Set up: 386-well plate (20 µl), Experiment type: MELT CURVE, Reagents used to detect target sequence: OTHER, Ramp Speed: STANDARD<br>(ii) Select the Define tab on the left, then select the following parameters: Target name: TARGET 1, Reporter: ROX, Quencher: NONE, Passive Reference: NONE<br>(iii) Select the Assign tab on the left, then perform the following actions: Highlight all 384 wells in the assay plate and check the box next to 'Target 1' on the top left of the plate layout. Note: check the 'Sample' box on the lower left.<br>(iv) Select the Run Method tab on the left, then make the following changes to the default Melt Curve profile: Delete Step 2 of the default cycle, change run method to "Step and Hold" with a 1:00 time, set the following temperatures: an initial 2:00 hold at 25 °C (4 °C), ramping up in increments of 1 °C to a final temperature of 95 °C (with a 2:00 hold), click on all three cameras to activate fluorescence detection throughout the experiment, and Select total volume per well of 20 µl<br>(v) Click on the RUN tab to the left to initiate thermal denaturation. |

**Supplementary Table 4**. Protocol for Firefly luciferase (FLuc) and NanoLuc (NLuc) enzymatic luminescence assay

**Supplementary Table 4:** Protocol for Firefly luciferase (FLuc) and NanoLuc (NLuc) enzymatic luminescence assay

Firefly luciferase (FLuc) enzymatic luminescence assay

| Sequence | Parameter | Value | Description |
|---|---|---|---|
| 1 | Reagent | 4 µL | No enzyme control (column 1) and enzyme solution (1-10 nM FLuc enzyme) in FLuc assay buffer; white/solid bottom, medium bind, high base plates (Greiner), dispensed with BioRaptr 2 |
| 2a | Compound Controls | 23 nL | DMSO vehicle control in columns 1-2; PTC124 32.8 µM in column 3 (high inhibition response) and PTC124 titrated 16-pt, 1:3, from 32.8 µM - 2.3 pM, in duplicate, in column 4; compounds transferred to respective columns with Pintool |
| 2b | Test Compounds | 23 nL | Test compounds: 10 mM stock concentration in 11-pt, 1:3 titration (32.8 µM – 0.56 nM final concentration range) in columns 5-48; compounds transferred to respective wells with Pintool |
| 3 | Incubation | 30 min | Incubate enzyme + compounds at ambient temp for 30 min, protected from light |
| 4 | Reagent | 3 µL | Add 3 µL of D-luciferin substrate solution to each well (final concentration 10 µM); dispensed with BioRaptr 2 |
| 5 | Detector | ViewLux | Read plate luminescence (Exposure = 1-10 sec; Gain = Medium-high; Speed = Slow; Binning = 2X) |

| Step | Notes |
|---|---|
| 1 | FLuc assay buffer: 66.6 mM Tris Acetate, pH 7.6; 10 mM $MgAc_2$; 0.01% Tween-20; 0.05% BSA; 10 µM ATP. All solutions filtered through 0.22 µm syringe filter prior to dispense. |
| 4 | D-Luciferin, CAS 2591-17-5 (Sigma, Cat # L9504) was prepared as a 10 mM stock solution in 100 mM Tris Acetate. Dispensing solution was diluted in 1X PBS, pH 7.4. |

NanoLuc luciferase (NLuc) enzymatic luminescence assay

| Sequence | Parameter | Value | Description |
|---|---|---|---|
| 1 | Reagent | 4 µL | No enzyme control (column 1) and enzyme solution (1 nM NLuc enzyme) in NLuc assay buffer; white/solid bottom, medium bind, high base plates (Greiner), dispensed with BioRaptr 2 |
| 2a | Compound Controls | 23 nL | DMSO vehicle control in columns 1-2; PTC124 3.3 µM in column 3, rows 1-16 and Cilnidipine 65.7 µM, rows 17-32 (high inhibition response), PTC124 titrated 8-pt, 1:5, from 3.3 µM - 42.1 pM, in duplicate, in column 4, rows 1-16 and Cilnidipine titrated 8-pt, 1:4, from 65.7 µM - 4.0 nM, in duplicate, in column 4, rows 17-32; compounds transferred to respective columns with Pintool |
| 2b | Test Compounds | 23 nL | Test compounds: 10 mM stock concentration in 11-pt, 1:3 titration (32.8 µM – 0.56 nM final concentration range) in columns 5-48; compounds transferred to respective wells with Pintool |
| 3 | Incubation | 30 min | Incubate enzyme + compounds at ambient temp for 30 min, protected from light |
| 4 | Reagent | 3 µL | Nano-Glo luciferase assay reagent (Promega) prepared according to manufacturer's protocol; dispensed with BioRaptr 2 |
| 5 | Detector | ViewLux | Read plate luminescence (Exposure = 1-10 sec; Gain = Medium-high; Speed = Slow; Binning = 2X) |

| Step | Notes |
|---|---|
| 1 | NLuc assay buffer: PBS, 0.05% IGEPAL; +/- 10 µM ATP. Solutions filtered through 0.22 µm syringe filter prior to dispense. |
| 4 | Nano-Glo luciferase assay reagent (Promega, Cat # N1120) prepared according to manufacturer's recommendations, components stored at -30 °C until use, and reagent prepared fresh for each assay. Reagent filtered through 0.22 µm syringe filter prior to dispense. |

**Supplementary Table 5**. Protocol for Abelson kinase (ABL1) and protein kinase A (PKA) peptide phosphorylation assays

**Supplementary Table 5:** Protocol for Abelson kinase (ABL1) and protein kinase A (PKA) peptide phosphorylation assays

| Sequence | Parameter | Value | Description |
|---|---|---|---|
| 1 | Reagent | 4 µL | No enzyme control (column 1) and enzyme solutions (17.5 nM ABL-*N*-HiBiT or PKA-*N*-HiBiT) in kinase assay buffer; white/solid bottom, medium bind, high base plates (Greiner), dispensed with BioRaptr 2 |
| 2a | Compound Controls | 25 nL | **ABL1**: DMSO vehicle control in columns 1-2; Imatinib 62.5 µM in column 3 (high inhibition response) and Imatinib titrated 16-pt, 1:2, from 62.5 µM - 1.9 nM, in duplicate, in column 4; compounds transferred to respective columns with Mosquito dispenser |
| | | | **PKA**: DMSO vehicle control in columns 1-2; H-89 titrated 16-pt, 1:2, from 62.5 µM - 1.9 nM, in duplicate, in columns 3-4; compounds transferred to respective columns with Mosquito Dispenser |
| 2b | Test Compounds | 23 nL | Test compounds: 10 mM stock concentration in 11-pt, 1:3 titration (57.5 µM – 37.4 nM final concentration range) in columns 5-48; compounds transferred to respective wells with Pintool |
| 3 | Incubation | 30 min | Incubate enzyme + compounds at ambient temp for 30 min, protected from light |
| 4 | Reagent | 25 nL | Peptide substrate (abltide, ABL1 or kemptide, PKA) was transferred to each well at a final concentration of 62.5 µM by Mosquito dispenser |
| 5 | Incubation | 3 hr | Ambient temperature, protected from light |
| 6 | Reagent | 4 µL | Kinase-Glo Plus reagent (Promega) prepared according to manufacturer's protocol; dispensed with BioRaptr 2 |
| 7 | Incubation | 10 min | Room temperature, protected from light |
| 8 | Detector | ViewLux | Read plate luminescence (Exposure = 1 sec; Gain = Medium-high; Speed = Slow; Binning = 2X) |

| Step | Notes |
|---|---|
| 1 | Kinase assay buffer: 20 mM Tris-HCl, pH 7.2; 5 mM $MgCl_2$; 150 mM KCl; 0.05% IGEPAL; 2 mM Dithiothreitol (DTT); 87.5 µM ATP. All solutions filtered through 0.22 µm syringe filter prior to dispense. |
| 4 | Peptide substrates prepared as 10 mM stocks in DMSO. |
| 6 | Kinase-Glo Plus reagent (Promega, Cat # V3771) prepared according to manufacturer's recommendations and stored at -30 °C until use. Reagent filtered through 0.22 µm syringe filter prior to dispense. |

**Supplementary Table 6**. Protocol for Co-factor independent phosphoglycerate mutase (iPGM) coupled-enzyme assay

**Supplementary Table 6:** Protocol for Co-factor independent phosphoglycerate mutase (iPGM) coupled-enzyme assay

| Sequence | Parameter | Value | Description |
|---|---|---|---|
| 1 | Reagent | 4 µL | No enzyme control (column 1) and enzyme solutions (5 nM Bm iPGM-*C*-HiBiT or Ce iPGM-*C*-HiBiT dialyzed against Zn/Mn final assay concentration) in PGM enzyme buffer; white/solid bottom, medium bind, high base plates (Greiner), dispensed with BioRaptr 2 |
| 2a | Compound Controls | 23 nL | DMSO vehicle control in columns 1-2; columns 3-4 Ce-2, Ce-2d, Sa-D2 and Sa-D3 control peptides +/- 19.2 µM 2-mercaptoethanol (BME) (Ce-2 and Ce-2d titrated from 3.8 µM – 49.1 pM in 8-pt 1:5 titration series in duplicate; Sa-D2 and Sa-D3 titrated from 19.2 µM – 68.5 pM in 8-pt 1:6 titration series in duplicate); compounds transferred to respective columns with Pintool |
| 2b | Test Compounds | 23 nL | Test compounds: macrocyclic peptides at 5 mM stock concentration in 16-pt, 1:3 titration (19.2 µM – 1.3 pM final concentration range) +/- 19.2 µM BME in columns 5-48; compounds transferred to respective columns with Pintool |
| 3 | Incubation | 20 min | Incubate enzyme + compounds at ambient temp for 20 min, protected from light |
| 4 | Reagent | 2 µL | Substrate solution; 0.4 mM final concentration 3PG in coupled-enzyme substrate buffer; dispensed with BioRaptr 2 |
| 5 | Incubation | 5 min | Ambient temperature, protected from light |
| 6 | Reagent | 4 µL | Kinase-Glo Plus reagent (Promega) prepared according to manufacturer's protocol; dispensed with BioRaptr 2 |
| 7 | Incubation | 10 min | Room temperature, protected from light |
| 8 | Detector | ViewLux | Read plate luminescence (Exposure = 1 sec; Gain = Medium; Speed = Slow; Binning = 2X) |

| Step | Notes |
|---|---|
| 1 | PGM enzyme buffer: 30 mM Tris-HCl, pH 8.0; 5 mM $MgSO_4$; 20 mM KCl; 0.12% BSA (0.08% final). All solutions filtered through 0.22 µm syringe filter prior to dispense. Ce iPGM-*C*-HiBiT enzyme dialyzed with Zn/Mn (6.63 µM stock solution in 20% glycerol); Bm iPGM-*C*-HiBiT enzyme 10 µM stock solution in 20% glycerol. |
| 4 | Coupled-enzyme substrate solution: 30 mM Tris-HCl, pH 8.0; 5 mM $MgSO_4$; 20 mM KCl; 9 mM ADP (3 mM final); 0.1 units/µl enolase (Millipore Sigma, Cat# E6126); 0.15 units/µl pyruvate kinase (from rabbit muscle, Millipore Sigma, Cat # P9136); 1.2 mM 3PG (0.4 mM final) (Millipore Sigma, Cat #P8877). Solution filtered through 0.22 µm syringe filter prior to dispense. |
| 6 | Kinase-Glo Plus reagent (Promega, Cat # V3771) prepared according to manufacturer's recommendations and stored at -30 °C until use. Reagent filtered through 0.22 µm syringe filter prior to dispense. |

**Supplementary Table 7**. Protocol for Co-factor independent phosphoglycerate mutase (iPGM) fluorescent polarization competition-biding assay

**Supplementary Table 7:** Protocol for *C. elegans* co-factor independent phosphoglycerate mutase (iPGM) fluorescent polarization competition-binding assay

| Sequence | Parameter | Value | Description |
|---|---|---|---|
| 1 | Reagent | 4 µL | No enzyme control (column 1) and enzyme solution (10 nM Ce iPGM-*C*-HiBiT dialyzed against Zn/Mn final assay concentration) in assay buffer + 0.05% IGEPAL; black/solid bottom, medium bind, high base plates (Greiner), dispensed with BioRaptr 2 |
| 2a | Compound Controls | 23 nL | DMSO vehicle control in columns 1-2; columns 3-4 Ce-2, Ce-2d, Sa-D2 and Sa-D3 control peptides +/- 19.2 µM 2-mercaptoethanol (BME) (Ce-2 and Ce-2d titrated from 3.8 µM – 49.1 pM in 8-pt 1:5 titration series in duplicate; Sa-D2 and Sa-D3 titrated from 19.2 µM – 68.5 pM in 8-pt 1:6 titration series in duplicate); compounds transferred to respective columns with Pintool |
| 2b | Test Compounds | 23 nL | Test compounds: macrocyclic peptides at 5 mM stock concentration in 16-pt, 1:3 titration (19.2 µM – 1.3 pM final concentration range) +/- 19.2 µM BME in columns 5-48; compounds transferred to respective columns with Pintool |
| 3 | Incubation | 20 min | Incubate enzyme + compounds at ambient temp for 20 min, protected from light |
| 4 | Pre-Read | Spark | Measure fluorescence polarization of fluorescein (X480, M535) on Spark multimode microplate reader |
| 5 | Reagent | 2 µL | Ce-2d-Fluorescein ligand (5 nM final concentration) in assay buffer + 0.05% IGEPAL; dispensed with BioRaptr 2 |
| 6 | Incubation | 30 min | Ambient temperature, protected from light |
| 7 | Detector | Spark | Measure fluorescence polarization of fluorescein (X480, M535) on Spark multimode microplate reader |

| Step | Notes |
|---|---|
| 1 | Assay buffer: 30 mM Tris-HCl, pH 8.0; 5 mM $MgSO_4$; 20 mM KCl; 0.05% IGEPAL. All solutions filtered through 0.22 µm syringe filter prior to dispense. Ce iPGM-*C*-HiBiT enzyme dialyzed with Zn/Mn (6.63 µM stock solution in 20% glycerol). |
| 4/7 | Measure fluorescence polarization of fluorescein on Spark multimode microplate reader with FP protocol: Excitation Wavelength [nm] “Monochrometer” = 485, BW = 20.0; Emission Wavelength [nm] “Monochrometer” = 535, BW = 20.0; G-Factor “Manual” = 1.000; Blank “Not Defined”. Advanced Settings: Flashes = 30; Gain “Optimal”; Mirror “Automatic”; Z-position [um] “Manual” = 20000; Settle Time [ms] = 0 |
| 5 | Fluorescence polarization ligand solution: 30 mM Tris-HCl, pH 8.0; 5 mM $MgSO_4$; 20 mM KCl; 0.05% IGEPAL; 15 nM macrocyclic peptide Ce-2d-Fluorescein (Ce-2d-Tyr11Lys-fluorescein) (5 nM final). Solution filtered through 0.22 µm syringe filter prior to dispense. |

**Supplementary Table 8**. Protocol for DNA ligase activity agarose gel assay

**Supplementary Table 8:** Protocol for DNA ligase activity agarose gel assay

| Sequence | Parameter | Value | Description |
|---|---|---|---|
| 1 | Reagent | 30 µL | Prepare ligase reactions with optimized ligase concentrations in appropriate buffer with Lamda/HindIII DNA substrate such that loading 30 µL of dye-quenched reaction mixture equates to 200 ng total DNA per well. Control ligase prepared according to manufacturor recommendation |
| 2 | Incubation | 60 min | Incubate ligase and DNA substrate at room temperature protected from light |
| 3 | Heat inactivation | 20 min | Heat inactivate all ligase reactions at 65 °C for 20 minutes. Chill briefly on ice, then centrifuge for 5 min at 12,000 g. |
| 3 | Reagent | 5.0 µL | Quench all ligase reactions by adding DNA loading dye (+SDS) |
| 4 | Gel Electrophoresis | 90 min | Load 30 µL of dye-quenched sample per lane of a 1% agarose gel (with GreenGlo Safe Fluorescent Dye). Resolve bands by gel electrophresis at 75V. |
| 5 | Detection | iBright | Detect DNA fluorescence using iBright nucleic-acid detection settings |
| **Step** | **Notes** | | |
| 1 | Enzymes were prepared at the following optimized conditions: | | *E. coli* Lig-*N*-HiBiT, 1.0 µM; 1X *E. coli* Ligase buffer (NEB) |
| | | | *E. coli* Lig-*C*-HiBiT, 100 nM; 1X *E. coli* Ligase buffer (NEB) |
| | | | T7 Lig-*N*-HiBiT, 100 nM; 1X StickTogether Buffer (NEB) |
| | | | T7 Lig-*C*-HiBiT, 1.0 µM; 1X StickTogether Buffer (NEB) |
| 4 | 1% agarose gel prepared using UltraPure Agarose (Invitrogen, Cat # 16500-100) dissolved in 1X TAE buffer. Molten agarose was supplimented with GreenGlo Safe DNA Dye (Denville Scientific) at 2 µL/100 mL. An equal concentration of dye was added to the 1X TAE running buffer. | | |

**Supplementary Table 9**. Protocol for Dihydrofolate reductase (DHFR) enzymatic absorbance assay

**Supplementary Table 9:** Protocol for Dihydrofolate reductase (DHFR) enzymatic absorbance assay

| Sequence | Parameter | Value | Description |
|---|---|---|---|
| 1 | Reagent | 4 µL | No enzyme control (column 1) and enzyme solution (100 nM DHFR-C-HiBiT final assay concentration) in 1X assay buffer + 75 µM NADPH; black/clear bottom, low base plates (Aurora), dispensed with BioRaptr 2 |
| 2a | Compound Controls | 23 nL | DMSO vehicle control in columns 1-2; column 3 Methotrexate competitor control 38.3 µM high concentration; column 4 Methotrexate titrated 16-pt, 1:3, from 38.3 µM - 2.7 pM, in duplicate; compounds transferred to respective columns with Pintool |
| 2b | Test Compounds | 23 nL | Test compounds: most at 10 mM high concentration in 11-pt, 1:3 titration (38.3 µM – 0.65 nM final concentration range) in columns 5-48; compounds transferred to respective wells with Pintool |
| 3 | Incubation | 20 min | Incubate enzyme + compounds at ambient temp for 20 min, protected from light |
| 4 | Pre-Read | Spark | Measure background absorbance at 340 nm on Spark multimode microplate reader |
| 5 | Reagent | 2 µL | Substrate solution; dihydrofolic acid (100 µM final concentration) in 1X assay buffer; dispensed with BioRaptr 2 |
| 6 | Incubation | 10 min | Ambient temperature, protected from light |
| 7 | Detector | Spark | Measure absorbance at 340 nm on Spark multimode microplate reader |

| Step | Notes |
|---|---|
| 1 | Assay buffer: 1X assay buffer diluted from 10X stock (Sigma-Aldrich, Cat # A5603); 75 µM NADPH. All solutions filtered through 0.22 µm syringe filter prior to dispense. |
| 4/7 | Measure absorbance at 340 nm on Spark multimode microplate reader with default reader settings: bandwidth = 5 nm, 25 flashes/well. |
| 5 | Dihydrofolic acid substrate solution: 1X assay buffer (Sigma-Aldrich, Cat # A5603); 300 µM dihydrofolic acid (100 µM final) (Sigma-Aldrich, Cat # D7006) prepared fresh according to manufacturer's protocol. Solution filtered through 0.22 µm syringe filter prior to dispense. |

**Supplementary Table 10**. Protocol for SDR assay non-aqueous ligand and aqueous ligand dispense

**Supplementary Table 10:** Protocol for SDR assay non-aqueous ligand and aqueous ligand dispense

SDR assay (non-aqueous ligand dispense)

| Sequence | Parameter | Value | Description |
|---|---|---|---|
| 1 | Reagent | 4 µL | No enzyme control (column 1) and enzyme solutions in SDR buffer (columns 2-48); white/solid bottom, medium bind, high base plates (Greiner), dispensed with BioRaptr 2 |
| 2a | Compound Controls | 23-25 nL | DMSO vehicle control in columns 1-2; columns 3-4 enzyme specific controls; compounds transferred to respective columns with Pintool or Mosquito dispenser |
| 2b | Test Compounds | 23 nL | Test compounds: 5-10 mM stock concentrations in 11- or 16-pt, 1:3 titration in columns 5-48; compounds transferred to respective wells with Pintool |
| 3 | Incubation | 30 min | Incubate enzyme + compounds at ambient temp for 30 min, protected from light |
| 4 | Reagent | 3 µL | Nano-Glo HiBiT lytic detection reagent (Promega) prepared according to manufacturer's protocol; dispensed with BioRaptr 2 |
| 5 | Incubation | 10 min | Room temperature, protected from light |
| 6 | Detector | ViewLux | Read plate luminescence (Exposure = 1 sec; Gain = Medium-high; Speed = Slow; Binning = 2X) |

| Step | Notes | |
|---|---|---|
| 1 | Enzymes were prepared at the following optimized conditions: | FLuc-*C*-NLuc, 1 nM final; 66.6 mM Tris Acetate, pH 7.6; 10 mM $MgAc_2$; 0.01% Tween-20; 0.05% BSA; ± 10 µM ATP |
| | | FLuc-N/C-HiBiT, 10 nM final; 66.6 mM Tris Acetate, pH 7.6; 10 mM MgAc2; 0.01% Tween-20; 0.05% BSA; ± 10 µM ATP |
| | | DHFR-*C*-HiBiT, 0.5 nM final; PBS, 0.03% IGEPAL, ± 2.9 µM NADPH |
| | | ABL1-*N*-HiBiT, 10 nM final; 11.4 mM Tris-HCl, pH 7.2, 2.8 mM $MgCl_2$, 85.4 mM KCl, 0.03% IGEPAL, 1.1 mM DTT |
| | | PKA-*N*-HiBiT, 10 nM final; 11.4 mM Tris-HCl, pH 7.2, 2.8 mM $MgCl_2$, 85.4 mM KCl, 0.03% IGEPAL, 1.1 mM DTT |
| | | iPGM-*C*-HiBiT, 0.5 - 5 nM final; 17.1 mM Tris-HCl, pH 8.0, 2.9 mM $MgSO_4$, 11.4 mM KCl, 0.03% IGEPAL |
| 2a | Compound controls were the same for each enzyme as those used in the functional enzymatic and FP assays (see supplementary tables 4-7) | |
| 4 | Nano-Glo HiBiT lytic detection reagent (Promega, Cat # N3040) prepared according to manufacturer's recommendations, components stored at -30 °C until use, and reagent prepared fresh for each assay. Reagent filtered through 0.22 µm syringe filter prior to dispense. | |
| 6 | NLuc luminescence read at the following optimized settings (Exposure; Gain; Speed; Binning): | FLuc-*C*-NLuc; 1 s; medium; slow; 2X |
| | | FLuc-*N*/*C*-HiBiT; 1 s; high; slow; 2X |
| | | DHFR-*C*-HiBiT; 1 s; medium; slow; 2X |
| | | ABL1-*N*-HiBiT; 1 s; high; slow; 2X |
| | | PKA-*N*-HiBiT; 1 s; medium; slow; 2X |
| | | iPGM-*C*-HiBiT; 1 s; medium (Bm iPGM) / high (Ce iPGM); slow; 2X |

SDR assay (aqueous ligand dispense)

| Sequence | Parameter | Value | Description |
|---|---|---|---|
| 1 | Reagent | 3 µL | No enzyme control (column 1) and enzyme solutions in 1.3x SDR buffer (columns 2-48, as required); white/solid bottom, medium bind, high base plates (Greiner), dispensed with Integra multi-channel auto pipettor |
| 2a | Compound Controls | 1 µL | Water vehicle control in columns 1-2 with Integra multi-channel auto pipettor |
| 2b | Test Compounds | 1 µL | Test compounds: 1-10 mM stock concentrations in 16-pt, 1:2 or 1:3 titration in columns 3-48 as required; compounds transferred to respective wells with Integra multi-channel auto pipettor |
| 3 | Incubation | 30 min | Incubate enzyme + compounds at ambient temp for 30 min, protected from light |
| 4 | Reagent | 3 µL | Nano-Glo HiBiT lytic detection reagent (Promega) prepared according to manufacturer's protocol; dispensed with BioRaptr 2 |
| 5 | Incubation | 10 min | Room temperature, protected from light |
| 6 | Detector | ViewLux | Read plate luminescence (Exposure = 1 sec; Gain = Medium-high; Speed = Slow; Binning = 2X) |

| Step | Notes | |
|---|---|---|
| 1 | Enzymes were prepared at the following optimized final conditions: | E. *Coli* Ligase-*N*/*C*-HiBiT, 0.5/1.0 nM final; 17.0 mM Tris-HCl, pH 8.0, 2.3 mM $MgCl_{2,}$ 50 µg/ml BSA, 1.1 mM DTT |
| | | T7 Ligase-*N*/*C*-HiBiT, 1.0 nM final; 37.7 mM Tris-HCl, pH 7.6, 5.7 mM $MgCl_{2,}$ 50 µg/ml BSA, 1.1 mM DTT |
| 2b | 22-mer dsDNA (1 mM) and ATP/NAD+ (10 mM) were dissolved in ultrapure water. Oligo DNA was titrated 16-pt, 1:3 in water in a 384-well storage plate and then transferred using manual 16-channel pipet in 1 uL. Cofactor ATP/NAD+ were titrated 16-pt 1:2 in water in a 384-well plate and transferred as described for DNA. | |
| 4 | Nano-Glo HiBiT lytic detection reagent (Promega, Cat # N3040) prepared according to manufacturer's recommendations, components stored at -30 °C until use, and reagent prepared fresh for each assay. Reagent filtered through 0.22 µm syringe filter prior to dispense. | |

**Supplementary Table 11**. SDR assay buffer and reader settings

| **Target**<br>protein-N/C-HiBiT / NLuc (concentration) | **SDR assay buffer (final)** | **Assay reader settings** (Exposure; Gain; Speed; Binning) | **Standardized basal RLU*** |
|---|---|---|---|
| FLuc-*C*-NLuc (1 nM) | 66.6 mM Tris Acetate, pH 7.6, 10 mM Magnesium Acetate, 0.01% Tween-20, 0.05% BSA ± 10 μM ATP | (1 s; medium; slow; 2X) | FLuc-*C*-NLuc + 10 μM ATP<br>6,311 ±214 |
| FLuc-*N*/*C*-HiBiT (10 nM) | 66.6 mM Tris Acetate, pH 7.6, 10 mM Magnesium Acetate, 0.01% Tween-20, 0.05% BSA ± 10 μM ATP | (1 s; high; slow; 2X) | FLuc-*N*-HiBiT + 10 μM ATP<br>689 ±24<br>FLuc-*C*-HiBiT + 10 μM ATP<br>1,049 ±69 |
| DHFR-*C*-HiBiT (0.5 nM) | PBS, pH 7.4, 0.03% IGEPAL ± 2.9 μM NADPH | (1 s; medium; slow; 2X) | DHFR-*C*-HiBiT + 2.9 μM NADPH<br>1,256 ±73 |
| ABL1-*N*/*C*-HiBiT (10 nM or 0.5 nM) | 11.4 mM Tris-HCl, pH 7.2, 2.8 mM $MgCl_2$, 85.4 mM KCl, 0.028% IGEPAL, 1.1 mM DTT | (1 s; high; slow; 2X) | ABL1-*N*-HiBiT<br>5,738 ± 167, 10 nM<br>ABL1-*C*-HiBiT<br>3,787 ± 150, 10 nM |
| PKA-*N*-HiBiT (10 nM) | 11.4 mM Tris-HCl, pH 7.2, 2.8 mM $MgCl_2$, 85.4 mM KCl, 0.028% IGEPAL, 1.1 mM DTT | (1 s; medium; slow; 2X) | PKA-*N*-HiBiT<br>15,154 ± 353 |
| *E. coli* Ligase (*N*-HiBiT 0.5 nM, *C*-HiBiT 1.0 nM) | 17 mM Tris-HCl, pH 8.0, 2.3 mM $MgCl_2$, 50 µg/ml BSA, 1.1 mM DTT | (1 s; medium; slow; 2X) | Ec-*N*-HiBiT<br>7,548 ± 751<br>Ec-*C*-HiBiT<br>13,715 ± 1127 |
| T7 Ligase-*N*/*C*-HiBiT (1.0 nM) | 37.7 mM Tris-HCl, pH 7.6, 5.7 mM $MgCl_2$, 50 µg/ml BSA, 1.1 mM DTT | (1 s; medium; slow; 2X) | T7-*N*-HiBiT<br>1,139 ± 157<br>T7-*C*-HiBiT<br>740 ± 49 |
| iPGM-*C*-HiBiT (0.5 - 5 nM) | 17.1 mM Tris-HCl, pH 8.0, 2.9 mM $MgSO_4$, 11.4 mM KCl, 0.03% IGEPAL | (1 s; medium (Bm iPGM)/high (Ce iPGM); slow; 2X) | Ce iPGM-*C*-HiBiT, 0.5 nM<br>474 ± 25<br>Bm iPGM-*C*-HiBiT, 5 nM<br>16,683 ± 993 |

*Standardized basal RLU references NLuc luminescence at optimal enzyme concentration with the following detector settings: 1 s exposure, medium gain, slow, 2X binning.

Source data are provided as a Source Data file.

**Supplementary Equations**.

Supplementary Equation (1) $coefficient\ of\ variation = \frac{\mu}{\sigma} \times 100$

Supplementary Equation (2) $signal - to - background = \frac{\sigma_p}{\sigma_n}$

Supplementary Equation (3) $signal - to - noise = \frac{\mu_p - \mu_n}{\sqrt{\sigma_p^2 + \sigma_n^2}}$

Supplementary Equation (4) $Z' = 1 - \frac{3(\sigma_p + \sigma_n)}{|\mu_p - \mu_n|}$

Supplementary Equation (5) $Y = Bottom + \frac{(Top - Bottom)}{1 + 10^{(Log\ EC50 - X) * Hill\ Slope}}$

Supplementary Equation (6) $Y = Bottom + \frac{(Top(1) - Bottom)}{1 + 10^{(Log\ EC50(1)\ -\ X) * Hill\ Slope}} + \frac{(Top(2) - Bottom)}{1 + 10^{(X -\ Log\ EC50(2)) * Hill\ Slope}}$

Supplementary Equation (7) $pIC_{50} = -log_{10} IC_{50}$

Supplementary Equation (8) $pSDR_{50} = -log_{10} \mathrm{SDR}_{50}$

* Where **σ** is the mean signal and **µ** is the standard deviation for the treated (p) or neutral (n) controls.

**Supplementary Figure 13a.** Uncropped Ponceau stained membrane

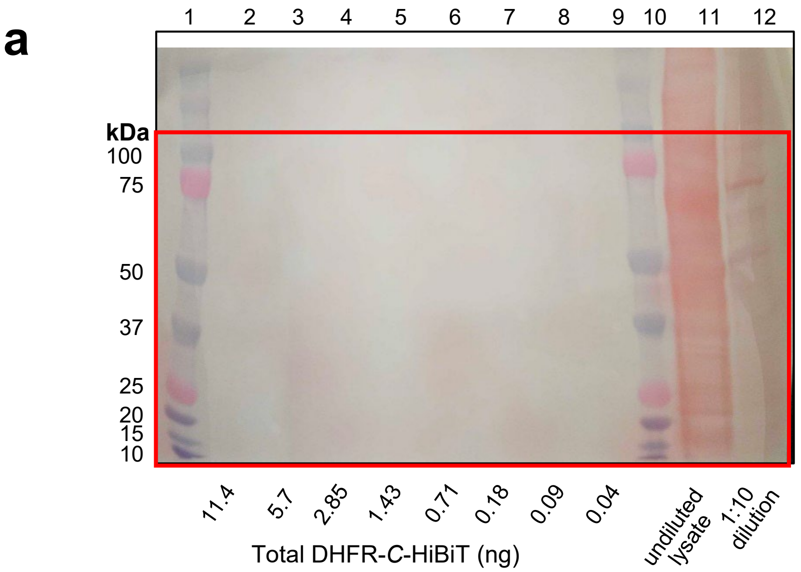

**Supplementary Figure 13b, c.** Uncropped anti-DHFR blot and Nanoblot

b

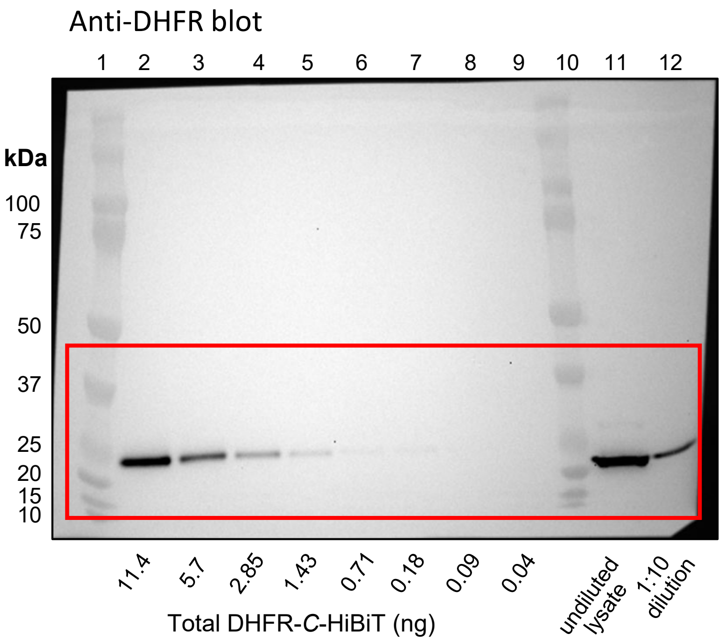


c

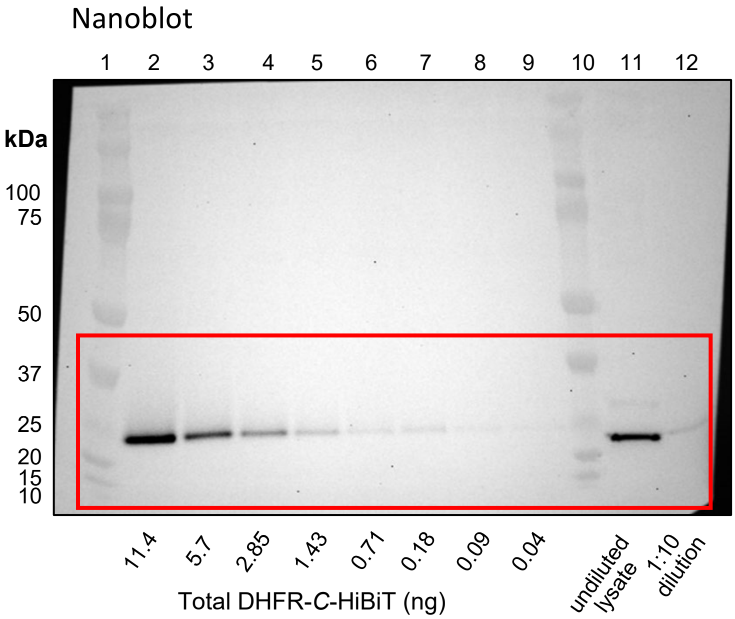

**Supplementary Figure 16**. Uncropped SDS-PAGE gel

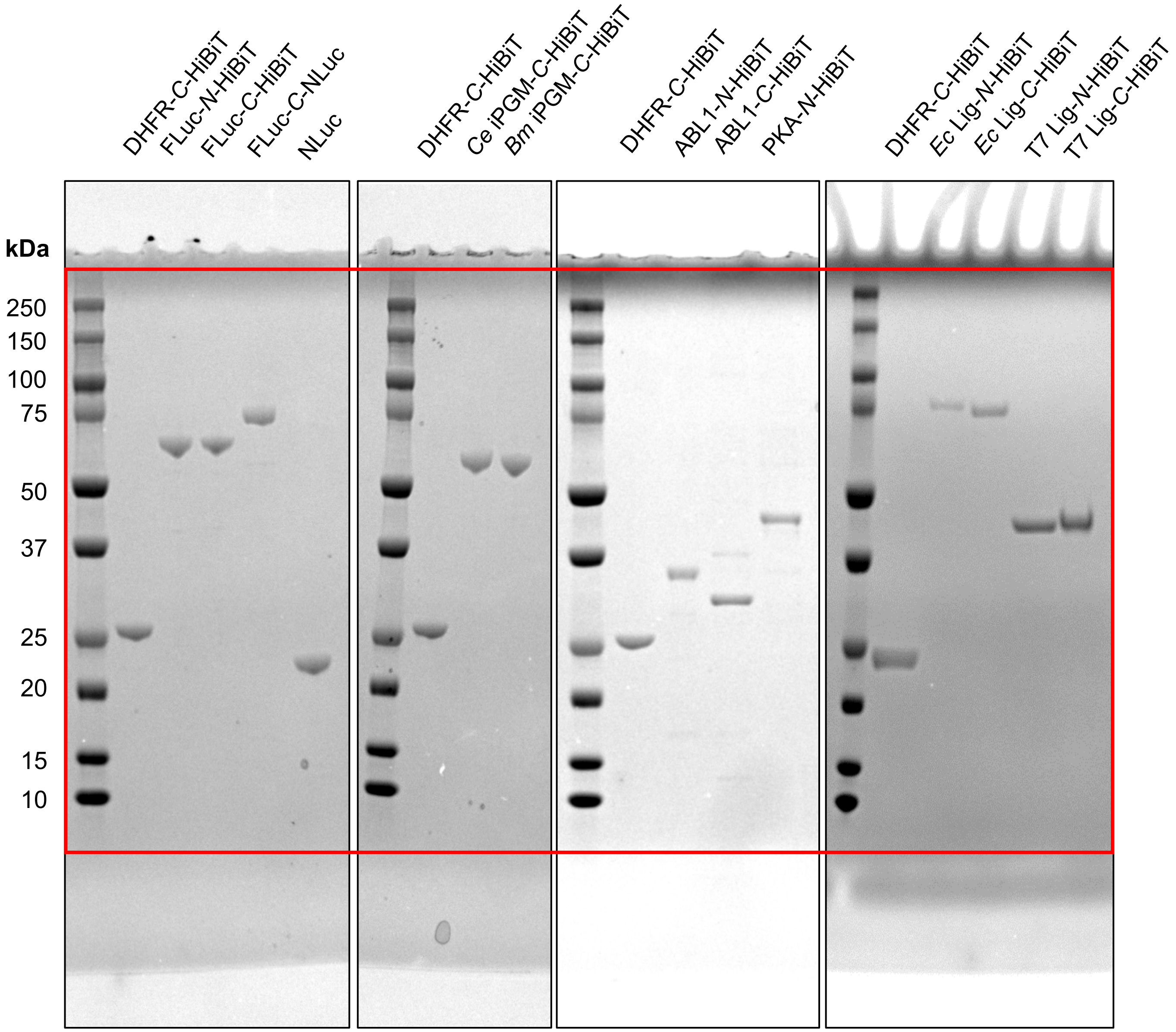

# **Supplementary Data.** Abltide HRMS analysis

## Qualitative Compound Report

| | | | |
|---|---|---|---|
| **Data File** | RHN001-016_1uLn01.d | **Sample Name** | RHN001-016 |
| **Sample Type** | Sample | **Position** | P1-D5 |
| **Instrument Name** | Instrument 1 | **User Name** | |
| **Acq Method** | WaterBEHcolumnPeptide100to3000mz8minGra_Profile.m | **Acquired Time** | 3/21/2024 1:10:23 PM |
| **IRM Calibration Status** | Success | **DA Method** | Formula confirmation_update03_peptide8mingrad2024March.m |
| **Comment** | 770.49 | | |

| | | | |
|---|---|---|---|
| **Sample Group** | | **Info.** | |
| **Forumla_1** | C32H62N14O8 | **Stream Name** | LC 1 |
| **Acquisition SW Version** | 6200 series TOF/6500 series Q-TOF B.06.01 (B6172 SP1) | | |

Fragmentor Voltage 175 Collision Energy 0 Ionization Mode ESI

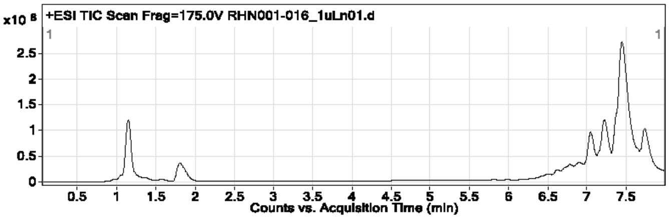


Fragmentor Voltage Collision Energy Ionization Mode

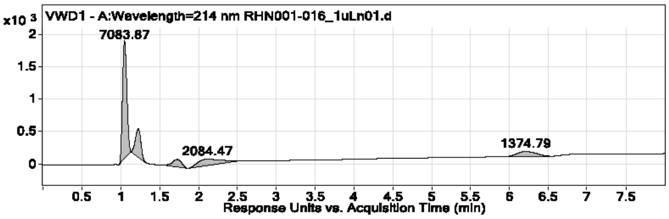


User Chromatogram Peak List

| RT | Height | Height % | Area | Area % | Area Sum % | Width |
|---|---|---|---|---|---|---|
| 1.047 | 1814.15 | 100 | 7083.87 | 100 | 52.06 | 0.153 |
| 1.223 | 433.12 | 23.87 | 2117.93 | 29.9 | 15.57 | 0.19 |
| 1.723 | 114.43 | 6.31 | 945.88 | 13.35 | 6.95 | 0.317 |
| 2.113 | 97.75 | 5.39 | 2084.47 | 29.43 | 15.32 | 0.68 |
| 6.21 | 77.38 | 4.27 | 1374.79 | 19.41 | 10.1 | 0.657 |

Compound Table

| Compound Label | RT | Mass | Abund | Formula | Tgt Mass | Diff (ppm) |
|---|---|---|---|---|---|---|
| Cpd 2: C32 H62 N14 O8 | 1.154 | 770.4785 | 3291724 | C32 H62 N14 O8 | 770.4875 | -11.73 |
| Cpd 1: C32 H62 N14 O8 | 1.8 | 770.4889 | 1718210 | C32 H62 N14 O8 | 770.4875 | 1.76 |

| Compound Label | *m/z* | RT | Algorithm | Mass |
|---|---|---|---|---|
| Cpd 2: C32 H62 N14 O8 | 386.2463 | 1.154 | Find By Formula | 770.4785 |

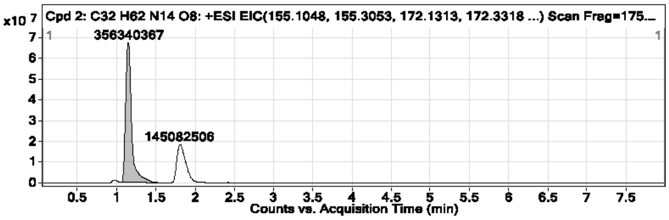


MS Spectrum

**Supplementary Data.** Abltide HRMS analysis

## Qualitative Compound Report

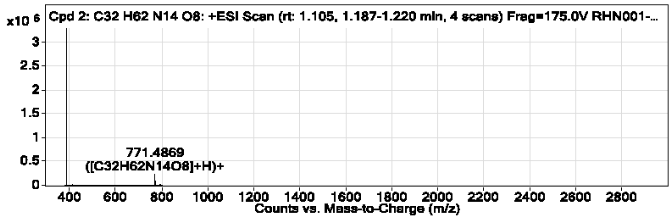


MS Zoomed Spectrum

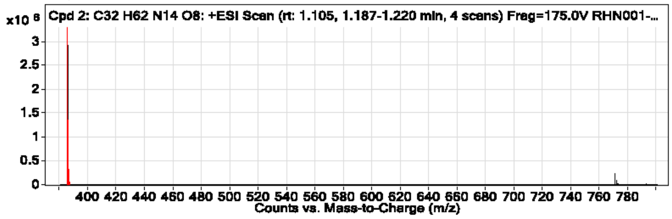


MS Spectrum Peak List

| m/z | Calc m/z | Diff(ppm) | z | Abund | Formula | Ion |
|---|---|---|---|---|---|---|
| 386.2463 | 386.251 | 12.23 | 2 | 3291723.86 | C32H62N14O8 | (M+2H)+2 |
| 386.7481 | 386.7523 | 10.91 | 2 | 1364591.82 | C32H62N14O8 | (M+2H)+2 |
| 387.2494 | 387.2536 | 10.72 | 2 | 331691.32 | C32H62N14O8 | (M+2H)+2 |
| 387.7497 | 387.7547 | 12.91 | 2 | 58300.27 | C32H62N14O8 | (M+2H)+2 |
| 403.3359 | 403.2776 | -144.59 | 2 | 63.25 | C32H62N14O8 | (M+2(NH4))+2 |
| 408.2288 | 408.233 | 10.28 | 2 | 4560.49 | C32H62N14O8 | (M+2Na)+2 |
| 771.4869 | 771.4948 | 10.2 | 1 | 229143.09 | C32H62N14O8 | (M+H)+ |
| 772.4893 | 772.4974 | 10.52 | 1 | 87474.69 | C32H62N14O8 | (M+H)+ |
| 788.4947 | 788.5213 | 33.72 | 1 | 94.01 | C32H62N14O8 | (M+NH4)+ |
| 793.4685 | 793.4767 | 10.41 | 1 | 20908.62 | C32H62N14O8 | (M+Na)+ |

| Compound Label | m/z | RT | Algorithm | Mass |
|---|---|---|---|---|
| Cpd 1: C32 H62 N14 O8 | 386.2516 | 1.8 | Find By Formula | 770.4889 |

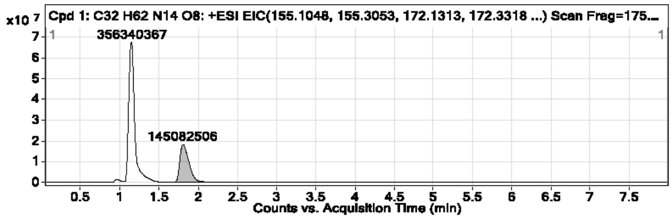


MS Spectrum

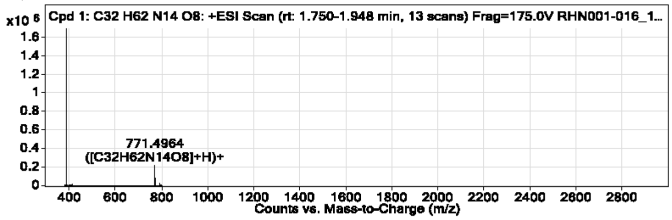


MS Zoomed Spectrum

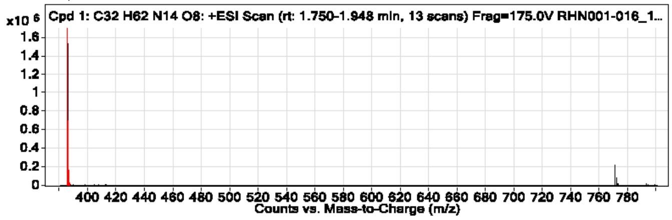


MS Spectrum Peak List

**Supplementary Data.** AbItide HRMS analysis

## Qualitative Compound Report

| *m/z* | *Calc m/z* | Diff(ppm) | z | Abund | Formula | Ion |
|---|---|---|---|---|---|---|
| 386.2516 | 386.251 | -1.38 | 2 | 1718210.16 | C32H62N14O8 | (M+2H)+2 |
| 386.7534 | 386.7523 | -2.72 | 2 | 679219.84 | C32H62N14O8 | (M+2H)+2 |
| 387.2543 | 387.2536 | -2 | 2 | 165340.35 | C32H62N14O8 | (M+2H)+2 |
| 387.7551 | 387.7547 | -1.02 | 2 | 27931.13 | C32H62N14O8 | (M+2H)+2 |
| 403.2126 | 403.2776 | 161.1 | 2 | 58.03 | C32H62N14O8 | (M+2(NH4))+2 |
| 408.2338 | 408.233 | -2.02 | 2 | 4031.02 | C32H62N14O8 | (M+2Na)+2 |
| 771.4964 | 771.4948 | -2.13 | 1 | 219738.72 | C32H62N14O8 | (M+H)+ |
| 772.4986 | 772.4974 | -1.51 | 1 | 81777.11 | C32H62N14O8 | (M+H)+ |
| 773.5001 | 773.4998 | -0.33 | 1 | 15955.36 | C32H62N14O8 | (M+H)+ |
| 793.4772 | 793.4767 | -0.61 | 1 | 20977.71 | C32H62N14O8 | (M+Na)+ |

--- End Of Report ---

# **Supplementary Data.** Kemptide HRMS analysis

## Qualitative Compound Report

| | | | |
|---|---|---|---|
| Data File | RHN001-018_1uL200uM01.d | Sample Name | RHN001-018 |
| Sample Type | Sample | Position | P1-D3 |
| Instrument Name | Instrument 1 | User Name | |
| Acq Method | WaterBEHcolumnPeptide100to3000mz8minGra_Profile.m | Acquired Time | 3/21/2024 10:16:18 AM |
| IRM Calibration Status | Success | DA Method | Formula confirmation_update03_peptide8mingrad2024March.m |
| Comment | 1263.7 | | |

| | | | |
|---|---|---|---|
| Sample Group | | Info. | |
| Forumla_1 | C60H93N15O15 | Stream Name | LC 1 |
| Acquisition SW | 6200 series TOF/6500 series | | |
| Version | Q-TOF B.06.01 (B6172 SP1) | | |

Fragmentor Voltage 175 Collision Energy 0 Ionization Mode ESI

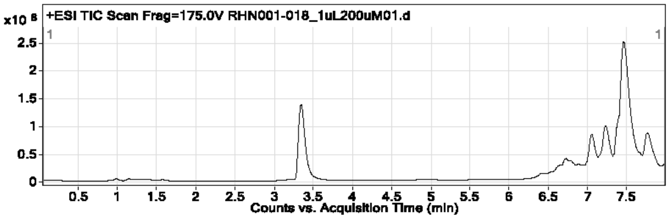


Fragmentor Voltage Collision Energy Ionization Mode

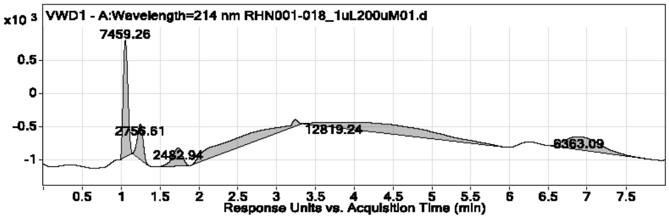


User Chromatogram Peak List

| RT | Height | Height % | Area | Area % | Area Sum % | Width |
|---|---|---|---|---|---|---|
| 1.05 | 1765.34 | 100 | 7459.26 | 58.19 | 17.85 | 0.167 |
| 1.243 | 537.31 | 30.44 | 2756.61 | 21.5 | 6.6 | 0.207 |
| 1.733 | 273.79 | 15.51 | 2482.94 | 19.37 | 5.94 | 0.437 |
| 3.24 | 104.62 | 5.93 | 9915.86 | 77.35 | 23.72 | 1.443 |
| 3.733 | 78.09 | 4.42 | 12819.24 | 100 | 30.67 | 2.617 |
| 6.89 | 198.83 | 11.26 | 6363.09 | 49.64 | 15.22 | 1.307 |

Compound Table

| Compound Label | RT | Mass | Abund | Formula | Tgt Mass | Diff (ppm) |
|---|---|---|---|---|---|---|
| Cpd 1: C60 H93 N15 O15 | 3.336 | 1263.7295 | 1061614 | C60 H93 N15 O15 | 1263.6976 | 25.26 |

| Compound Label | *m/z* | RT | Algorithm | Mass |
|---|---|---|---|---|
| Cpd 1: C60 H93 N15 O15 | 422.2509 | 3.336 | Find By Formula | 1263.7295 |

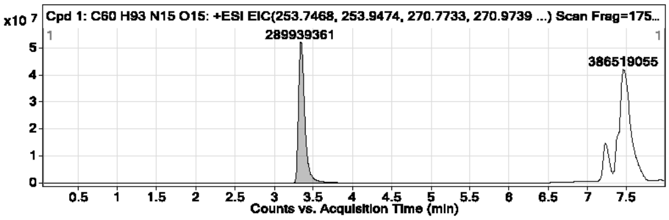


MS Spectrum

**Supplementary Data.** Kemptide HRMS analysis

## Qualitative Compound Report

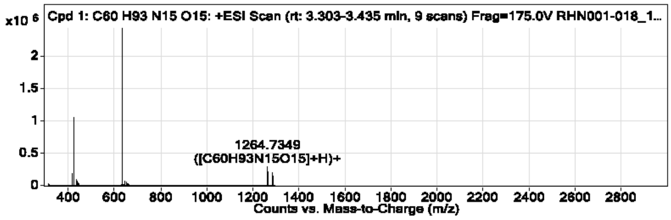


MS Zoomed Spectrum

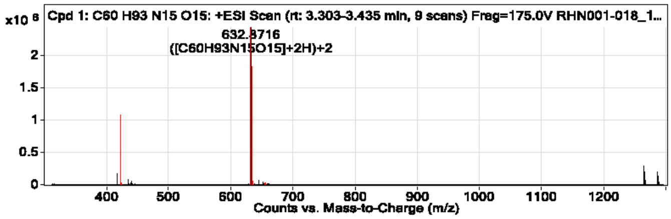


MS Spectrum Peak List

| *m/z* | *Calc m/z* | Diff(ppm) | z | Abund | Formula | Ion |
|---|---|---|---|---|---|---|
| 316.8795 | 316.9317 | 164.74 | 4 | 112.35 | C60H93N15O15 | (M+4H)+4 |
| 422.2509 | 422.2398 | -26.22 | 3 | 1061613.91 | C60H93N15O15 | (M+3H)+3 |
| 439.2223 | 439.2663 | 100.37 | 3 | 1855.28 | C60H93N15O15 | (M+3(NH4))+3 |
| 444.2645 | 444.2217 | -96.32 | 3 | 3939.28 | C60H93N15O15 | (M+3Na)+3 |
| 632.8716 | 632.8561 | -24.58 | 2 | 2496510.37 | C60H93N15O15 | (M+2H)+2 |
| 649.8312 | 649.8826 | 79.04 | 2 | 246.79 | C60H93N15O15 | (M+2(NH4))+2 |
| 654.8534 | 654.838 | -23.44 | 2 | 26175.44 | C60H93N15O15 | (M+2Na)+2 |
| 1264.7349 | 1264.7048 | -23.79 | 1 | 289956.81 | C60H93N15O15 | (M+H)+ |
| 1281.6731 | 1281.7314 | 45.47 | 1 | 89.95 | C60H93N15O15 | (M+NH4)+ |
| 1286.7171 | 1286.6868 | -23.53 | 1 | 196472.47 | C60H93N15O15 | (M+Na)+ |

--- End Of Report ---

**Supplementary Data.** Ce-2d K11-FL (GRB063-038) HRMS analysis

# Qualitative Compound Report

| | | | |
|---|---|---|---|
| Data File | GRB063-038_1uL001.d | Sample Name | CM-GRB063-038 |
| Sample Type | Sample | Position | P1-F4 |
| Instrument Name | Instrument 1 | User Name | |
| Acq Method | RaulRP182peptide100to3000mz8minGra_profileHALO_REF.m | Acquired Time | 4/21/2025 12:23:44 PM |
| IRM Calibration Status | Success | DA Method | Formula confirmation_peptide_2024Aug.m |
| Comment | | | |

| | | | |
|---|---|---|---|
| Sample Group | | Info. | |
| Formula | C89H97N13O26S | Exact MS | 1795.6 |
| Stream Name | LC 1 | Acquisition SW | 6200 series TOF/6500 series |
| | | Version | Q-TOF B.06.01 (B6157) |

Fragmentor Voltage 175 Collision Energy 0 Ionization Mode ESI

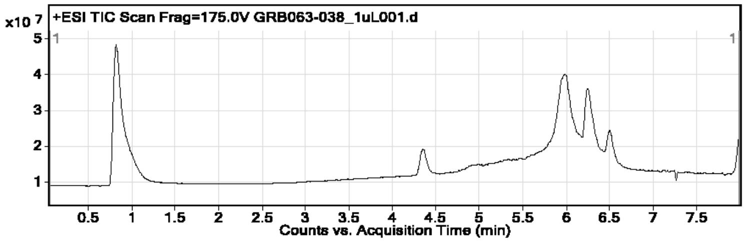


Fragmentor Voltage Collision Energy Ionization Mode

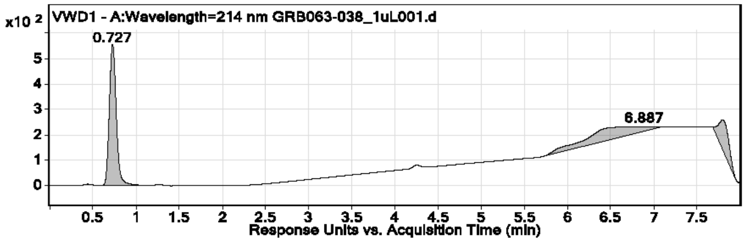


**User Chromatogram Peak List**

| RT | Height | Height % | Area | Area % | Area Sum % | Width |
|---|---|---|---|---|---|---|
| 0.727 | 556 | 100 | 3099.68 | 100 | 50.21 | 0.593 |
| 6.887 | 18.36 | 3.3 | 2108.1 | 68.01 | 34.15 | 1.387 |
| 7.8 | 118.33 | 21.28 | 965.36 | 31.14 | 15.64 | 0.267 |

**Compound Table**

| Compound Label | RT | Mass | Abund | Formula | Tgt Mass | Diff (ppm) |
|---|---|---|---|---|---|---|
| Cpd 1: C89 H97 N13 O26 S | 4.349 | 1795.6417 | 1339 | C89 H97 N13 O26 S | 1795.6388 | 1.6 |

| Compound Label | *m/z* | RT | Algorithm | Mass |
|---|---|---|---|---|
| Cpd 1: C89 H97 N13 O26 S | 599.554 | 4.349 | Find By Formula | 1795.6417 |

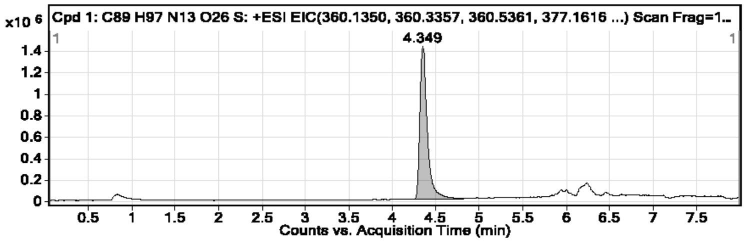


MS Spectrum

**Supplementary Data.** Ce-2d K11-FL (GRB063-038) HRMS analysis

## Qualitative Compound Report

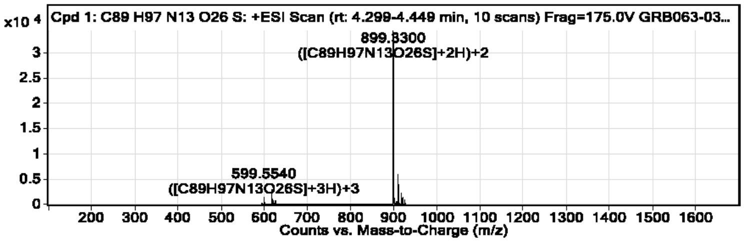


MS Zoomed Spectrum

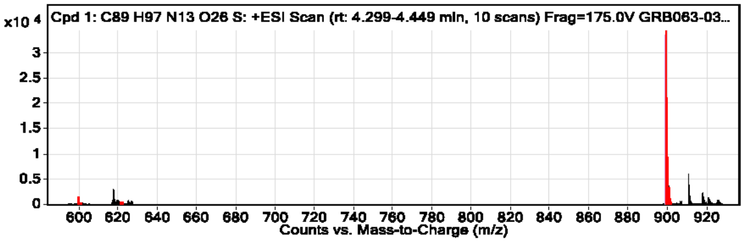


**MS Spectrum Peak List**

| *m/z* | *Calc m/z* | Diff(ppm) | z | Abund | Formula | Ion |
|---|---|---|---|---|---|---|
| 599.554 | 599.5536 | -0.76 | 3 | 1339.49 | C89H97N13O26S | (M+3H)+3 |
| 599.8886 | 599.8879 | -1.12 | 3 | 1260.63 | C89H97N13O26S | (M+3H)+3 |
| 621.5302 | 621.5355 | 8.54 | 3 | 386.81 | C89H97N13O26S | (M+3Na)+3 |
| 899.33 | 899.3282 | -2.01 | 2 | 34673.46 | C89H97N13O26S | (M+2H)+2 |
| 899.8313 | 899.8293 | -2.23 | 2 | 21220.5 | C89H97N13O26S | (M+2H)+2 |
| 900.3316 | 900.3303 | -1.5 | 2 | 9387.31 | C89H97N13O26S | (M+2H)+2 |
| 900.8332 | 900.8312 | -2.2 | 2 | 3622.7 | C89H97N13O26S | (M+2H)+2 |
| 901.3347 | 901.3321 | -2.81 | 2 | 919.17 | C89H97N13O26S | (M+2H)+2 |
| 915.8242 | 915.8532 | 31.72 | 2 | 90.7 | C89H97N13O26S | (M+2(NH4))+2 |
| 921.3115 | 921.3102 | -1.46 | 2 | 1364.72 | C89H97N13O26S | (M+2Na)+2 |

--- End Of Report ---

**HRMS analysis**. Peptide samples were subjected to HPLC-TOF MS analysis. Liquid chromatography (LC) was performed on an Agilent 1290 Infinity II LC system (Agilent Technologies, Wilmington, DE, U.S.A.) equipped with a diode array detector (DAD), binary pump, multicolumn thermostat, and autosampler. The mobile phases used for the separation were MS-grade water with 0.1% formic acid (solvent A) and MS-grade acetonitrile with 0.1% formic acid (solvent B). Gradient elution of peptides from the analytical column (The ACQUITY UPLC BEH C18 column, 2.1×100 mm, particle size 1.7 μm) was performed using a gradient starting at 5% B at a flow rate of 0.4 mL/min. The mobile phase was then 5–15% B for 1 min, 15-45%B for 3.5 min, 45 to 90%B for 0.5 min and maintained at 90% B for 1.5 min, followed by 5% B for 0.5 min and re-equilibration of the column with 5% B for 1.4 min. Separations were performed at a column temperature of 60 °C with a total run time of 8 min. Mass spectrometry (MS) experiments were conducted on an Agilent 6230 TOF system (Agilent Technologies, Wilmington, DE, U.S.A.), equipped with a DUAL AJS ESI source operating in positive ion mode. MS spectra were acquired from m/z 300 to 3000 at a scan rate of 1 spectrum per second with Profile format. The electrospray ionization (ESI) source parameters were used as follows: gas temperature, 325 °C; gas flow, 8 L/min; nebulizer, 35 psi; sheath gas temperature: 350 °C; Vcap, 3500 V; nozzle,1000 V; fragmentor, 175 V. HPLC-TOF data analysis was performed by Agilent MassHunter Qualitative Analysis (B.07.00).
