# Supplementary material for: A general assay platform to study protein pharmacology using ligand-dependent structural dynamics
Source: Nat Commun. 2025 May 10;16:4342. doi: 10.1038/s41467-025-59658-6 (PMC12064818; doi:10.1038/s41467-025-59658-6)
Supplement: Supplementary file 2 — Description of Additional Supplementary Files [file 41467_2025_59658_MOESM2_ESM.pdf]

Description of Additional Supplementary Files

File Name: Supplementary Data 1
Description: Firefly luciferase (FLuc) inhibitor library

File Name: Supplementary Data 2
Description: FLuc-C-HiBiT qHTS results

File Name: Supplementary Data 3
Description: Clade S follow-up results

File Name: Supplementary Data 4
Description: 128-member ABL1 kinase inhibitor-enriched library

File Name: Supplementary Data 5
Description: ABL1-N-HiBiT qHTS results

File Name: Supplementary Data 6
Description: PKA-N-HiBiT qHTS results

File Name: Supplementary Data 7
Description: Standard and SDR assay statistics

File Name: Supplementary Data 8
Description: Control condition layout for 1536-well assay plates
