# Supplementary material for: A general assay platform to study protein pharmacology using ligand-dependent structural dynamics
Source: Nat Commun. 2025 May 10;16:4342. doi: 10.1038/s41467-025-59658-6 (PMC12064818; doi:10.1038/s41467-025-59658-6)
Supplement: Supplementary file 11 — Reporting Summary [file 41467_2025_59658_MOESM11_ESM.pdf]

Corresponding author(s): James Inglese

Last updated by author(s): Mar 5, 2025

Reporting Summary

Nature Portfolio wishes to improve the reproducibility of the work that we publish. This form provides structure for consistency and transparency in reporting. For further information on Nature Portfolio policies, see our Editorial Policies and the Editorial Policy Checklist.

Statistics

For all statistical analyses, confirm that the following items are present in the figure legend, table legend, main text, or Methods section.

n/a	Confirmed	
☐	☒	The exact sample size (*n*) for each experimental group/condition, given as a discrete number and unit of measurement
☐	☒	A statement on whether measurements were taken from distinct samples or whether the same sample was measured repeatedly
☐	☒	The statistical test(s) used AND whether they are one- or two-sided<br>*Only common tests should be described solely by name; describe more complex techniques in the Methods section.*
☒	☐	A description of all covariates tested
☐	☒	A description of any assumptions or corrections, such as tests of normality and adjustment for multiple comparisons
☐	☒	A full description of the statistical parameters including central tendency (e.g. means) or other basic estimates (e.g. regression coefficient) AND variation (e.g. standard deviation) or associated estimates of uncertainty (e.g. confidence intervals)
☐	☒	For null hypothesis testing, the test statistic (e.g. *F*, *t*, *r*) with confidence intervals, effect sizes, degrees of freedom and *P* value noted<br>*Give P values as exact values whenever suitable.*
☒	☐	For Bayesian analysis, information on the choice of priors and Markov chain Monte Carlo settings
☐	☒	For hierarchical and complex designs, identification of the appropriate level for tests and full reporting of outcomes
☐	☒	Estimates of effect sizes (e.g. Cohen's *d*, Pearson's *r*), indicating how they were calculated

Our web collection on statistics for biologists contains articles on many of the points above.

Software and code

Policy information about availability of computer code

Data collection	Data collection software used in this study included a ViewLux CCD imager (PerkinElmer) for luminescence measurements , a Spark multimode microplate reader (Tecan) for fluorescence polarization, absorbance, and BCA protein measurements, and an iBright gel imager (ThermoFisher Scientific) for gel and Western blot analysis.
Data analysis	Concentration response curves and correlation plots were analyzed in Prism 8 (GraphPad software, version 8.1.3). Compound selection for library construction was performed using PubChem data and a Pharos annotated list of kinase inhibitors. 3-axis plots were generated with 3D qHTS waterfall plot software (https://github.com/ncats/qHTSWaterfall). TIBCO Spotfire with Perkin-Elmer Lead Discovery was used UMPGA hierarchical clustering. In house software was used to fit the inter-plate or intra-plate titration data with a standard Hill equation (Wang et al. 2010).

For manuscripts utilizing custom algorithms or software that are central to the research but not yet described in published literature, software must be made available to editors and reviewers. We strongly encourage code deposition in a community repository (e.g. GitHub). See the Nature Portfolio guidelines for submitting code & software for further information.

Data

Policy information about availability of data

All manuscripts must include a data availability statement. This statement should provide the following information, where applicable:

- Accession codes, unique identifiers, or web links for publicly available datasets
- A description of any restrictions on data availability
- For clinical datasets or third party data, please ensure that the statement adheres to our policy

All data generated in this study is freely available in the supplementary data files, source data file and/or deposited in PubChem with AIDs 1963320; 1963319; 1963318; 1963317; 1963315; 1963321; 1963322

Research involving human participants, their data, or biological material

Policy information about studies with human participants or human data. See also policy information about sex, gender (identity/presentation), and sexual orientation and race, ethnicity and racism.

Reporting on sex and gender	N/A
Reporting on race, ethnicity, or other socially relevant groupings	N/A
Population characteristics	N/A
Recruitment	N/A
Ethics oversight	N/A

Note that full information on the approval of the study protocol must also be provided in the manuscript.

Field-specific reporting

Please select the one below that is the best fit for your research. If you are not sure, read the appropriate sections before making your selection.

☒ Life sciences ☐ Behavioural & social sciences ☐ Ecological, evolutionary & environmental sciences

For a reference copy of the document with all sections, see nature.com/documents/nr-reporting-summary-flat.pdf

Life sciences study design

All studies must disclose on these points even when the disclosure is negative.

Sample size	Z-factor analysis was used as described in the paper to confirm sample sizes gave statistically significant signal to background and variation coefficients for the assays described in this study. Experimental sample sizes are described in respective figure legends.
Data exclusions	Data were only excluded when there were obvious automated dispense error of reagent, i.e. enzyme, protein, substrate, or detection reagent were not dispensed into a given 1536-well microtiter plate assay well. Wells were only excluded when the luminescence RLU value was at no enzyme background levels.
Replication	All experiments were conducted as two or more replicates, with the exception of the large-scale FLuc library qHTS (1,343 compounds) which was done once, however all compound samples are essentially replicated as an 11 point titration.
Randomization	The distribution of library compounds were randomly assigned to plates. However, for all subsequent experiments, randomization is generally not required as independent variables are controlled for in the experimental design, for example, by including DMSO vehicle wells or plates to allow for correction and normalization of plate field variations that could arise from, for example plate edge effects. The use of full compound titrations at all stages of this work further reduces potential for random variation affecting the objective outcomes of these data.
Blinding	qHTS is inherently blinded as the location of compound titrations on or through any given plate are numerous and not known until data analysis and the outcome, a concentration response curve, is an objective measure of the experimental treatment and measurement.

Reporting for specific materials, systems and methods

We require information from authors about some types of materials, experimental systems and methods used in many studies. Here, indicate whether each material, system or method listed is relevant to your study. If you are not sure if a list item applies to your research, read the appropriate section before selecting a response.

Materials & experimental systems

n/a	Involved in the study
☐	☒ Antibodies
☐	☒ Eukaryotic cell lines
☒	☐ Palaeontology and archaeology
☒	☐ Animals and other organisms
☒	☐ Clinical data
☒	☐ Dual use research of concern
☒	☐ Plants

Methods

n/a	Involved in the study
☒	☐ ChIP-seq
☒	☐ Flow cytometry
☒	☐ MRI-based neuroimaging

Antibodies

Antibodies used

Recombinant Anti-Dihydrofolate reductase (DHFR) antibody [EPR5285] (ab124814) in rabbit (RRID:AB_10975115); , anti-rabbit IgG HRP (Invitrogen, Cat # A16096; polyclonal secondary; RRID:AB_2534770)

Validation

Commercially validated and available from Abcam (https://www.abcam.com/en-us/products/primary-antibodies/dihydrofolate-reductase-dhfr-antibody-epr5285-ab124814), Invitrogen (https://www.thermofisher.com/antibody/product/Goat-anti-Rabbit-IgG-H-L-Secondary-Antibody-Polyclonal/A16096)

Eukaryotic cell lines

Policy information about cell lines and Sex and Gender in Research

Cell line source(s)

HEK293 cell line constitutively expressing S. pyogenes Cas9 from GeneCopoeia, Cat # SL553

Authentication

Cell identity confirmed by vendor (GeneCopoeia) and genome edited insert confirmed by Western blot (Supplemenatary figure 13).

Mycoplasma contamination

Cell cultures were routinely tested for Mycoplasma contamination using the MycoAlert PLUS Mycoplasma Detection Kit (Lonza Bioscience, cat # LT07) according to manufacturer protocol. Cells in this study tested negative for Mycoplasma.

Commonly misidentified lines
(See ICLAC register)

N/A

Plants

Seed stocks

N/A

Novel plant genotypes

N/A

Authentication

N/A
